# Supplementary material for: Stem girth changes in response to soil water potential in lowland dipterocarp forest in Borneo: An individualistic time-series analysis
Source: PLoS One. 2022 Jun 30;17(6):e0270140. doi: 10.1371/journal.pone.0270140 (PMC9246238; doi:10.1371/journal.pone.0270140)

S1 Appendix: Table A. Details of the 18 selected trees and their electronic bands (eDDS); with species codes, reference tags, three measures of canopy exposure (layer, space and light level – defined in the footnotes), these last averaged into a canopy status (canStat) value, and relative growth rates (Rgr) from main plot census P_3, 2001-2007 (*rgr*) for the wet and dry periods.

			Canopy				Rgr in P_3
Band	Tag	Code	Layer[1]	Space[2]	Light[3]	CanStat	(mm/m/yr)
g11	560	Mw	2	2	2	2.00	14.01
g12	567	Sf	4	4.5	5	4.50	49.70
g14	577	Lb	2.5	3	3	2.83	20.62
g15	1084	Sf	2	3	1.5	2.17	10.84
g22	1662	Pm	3.5	3.5	3.5	3.50	8.96
g23	1732	Dm	2	3	2	2.33	9.36
g24	1685	Mw	2	3	3	2.67	10.69
g25	2405	Sf	2	2	2.5	2.17	23.85
g31	7680	Mw	2.5	2	2	2.17	9.33
g32	6892	Sp	4	4	4.5	4.17	55.42
g33	6900	Lb	3	2.5	2.5	2.67	33.91
g34	7436	Dm	2	2.5	3	2.50	2.11
g35	7586	Dm	2	3	1.5	2.17	0.00
g41	8077	Sf	2	2.5	2	2.17	0.00
g42	8060	Dm	2.5	2	1.5	2.00	0.00
g43	8321	Pm	4	4	4	4.00	12.30
g44	8848	Sp	4	4	4.5	4.17	18.94
g45	8810	Pm	3	4	3.5	3.50	8.92

[1] Layer of tree: 1 tree-let; 2 understorey; 3 mid-level; 4 top canopy; 5 emergent. [2] Space of crown: 1, completely suppressed; 2, mixed/entangled from all sides; 3, significantly overlapping most sides; 4, stems touching from some sides or above; 5, crown free, unshaded; by neighbours. [3] Light level: 1 < 30%; 2, 30 - < 50%; 3, 50 - < 70%; 4, 70 - < 90; 5, ≥ 90% of crown receiving full light

S1 Appendix: Table B. **Means, with standard deviations and lower and upper quartiles, of the 1-day girth increment, *gthi*, for the wet (n = 550) and dry (n = 93-98; see text) periods.** Values are *gthi* in cm x 1000; sd is standard deviation; q1 and q3 are lower and upper quartiles. Codex, are the codes in Table 2 of the main text, with individuals within species numbered.

nr	station	tree	Codex	band	wet				dry			
					mean	sd	q1	q3	mean	sd	q1	q3
1	1	1	Mw1	g11	0.589	9.681	−4.0	6.5	1.623	8.564	−2.9	5.5
2	1	2	Sf1	g12	8.320	30.850	−9.6	23.2	1.128	4.692	−2.1	4.6
3	1	4	Lb1	g14	0.540	23.500	−4.9	6.0	0.770	13.110	−6.4	2.8
4	1	5	Sf2	g15	−0.451	14.875	−9.4	9.5	−1.480	14.950	−12.0	8.3
5	2	2	Pm1	g22	0.840	23.860	−5.0	5.4	−0.628	8.915	−6.3	3.1
6	2	3	Dm1	g23	0.097	9.347	−4.0	4.6	0.210	6.591	−4.6	5.0
7	2	4	Mw2	g24	−0.170	4.264	−2.7	1.9	1.369	8.674	−3.3	4.4
8	2	5	Sf3	g25	−0.240	10.505	−6.0	6.5	−0.560	11.560	−7.1	5.2
9	3	1	Mw3	g31	−0.057	4.655	−2.7	2.3	2.450	18.180	−5.6	6.9
10	3	2	Sp1	g32	7.397	10.933	0.6	14.6	5.996	5.298	3.0	10.2
11	3	3	Lb2	g33	2.470	4.047	−0.2	4.6	3.571	7.780	−1.3	7.4

12	3	4	Dm2	g34	−0.240	4.919	−3.3	2.7	0.929	5.310	−3.0	5.2
13	3	5	Dm3	g35	0.035	13.139	−5.6	6.6	0.277	7.099	−4.0	5.6
14	4	1	Sf4	g41	−0.090	3.493	−1.9	1.7	–	–	–	–
15	4	2	Dm4	g42	0.001	4.024	−2.3	2.3	–	–	–	–
16	4	3	Pm2	g43	3.831	22.445	−8.1	13.1	–	–	–	–
17	4	4	Sp2	g44	3.641	9.459	−1.9	8.7	–	–	–	–
18	4	5	Pm3	g45	−0.933	10.674	−6.3	3.7	–	–	–	–

S1 Appendix: Table C. Mean and SEs of the soil moisture potential (SMP with lag 1 d) and logger temperature (no lag) coefficients from the finally selected GLS time-series regressions, that are plotted in Figs. 4 and 5. Here ‘code’ in Table 3 of the main text is expanded to ‘codex’ to show the tree number per species as labelled on the Figs 6-10. Note: the coefficients are not here standardized for girth, as they are in main text Figs 6-10.

number	station	tree	codex	gth	SMP_{-1} wet		$TEMP_0$ wet		SMP_{-1} dry		$TEMP_0$ dry	
					est	se	est	se	est	se	est	se
1	1	1	Mw1	g11	1.517	0.218	0.945	0.310	-0.103	0.125	0.230	0.439
2	1	2	Sf1	g12	−4.688	0.923	−5.249	0.915	-0.026	0.096	-1.176	0.267
3	1	4	Lb1	g14	−0.046	0.431	−0.736	0.504	-0.617	0.245	2.882	0.674
4	1	5	Sf2	g15	2.297	0.345	2.182	0.457	1.298	0.247	0.900	0.706
5	2	2	Pm1	g22	−1.531	0.221	2.219	0.365	-0.155	0.141	2.805	0.424
6	2	3	Dm1	g23	0.934	0.249	1.587	0.294	0.514	0.155	-1.028	0.390
7	2	4	Mw2	g24	−0.166	0.078	0.574	0.130	-0.425	0.111	1.803	0.402
8	2	5	Sf3	g25	1.517	0.245	1.997	0.316	0.893	0.126	1.460	0.629
9	3	1	Mw3	g31	−0.235	0.056	0.584	0.120	-0.897	0.218	3.567	0.880
10	3	2	Sp1	g32	−0.094	0.320	0.504	0.339	0.549	0.091	0.274	0.309
11	3	3	Lb2	g33	−0.455	0.104	0.862	0.136	-0.197	0.174	-1.905	0.466
12	3	4	Dm2	g34	−0.430	0.100	0.141	0.158	-0.362	0.113	-0.134	0.247
13	3	5	Dm3	g35	1.248	0.291	2.553	0.419	0.287	0.095	0.230	0.439
14	4	1	Sf4	g41	−0.271	0.094	0.619	0.181	–	–	–	–
15	4	2	Dm4	g42	0.504	0.073	1.055	0.138	–	–	–	–
16	4	3	Pm2	g43	−2.825	0.480	2.111	0.693	–	–	–	–
17	4	4	Sp2	g44	−2.291	0.277	0.567	0.302	–	–	–	–
18	4	5	Pm3	g45	−1.205	0.221	0.480	0.307	–	–	–	–

S1 Appendix: Table D. *F*-ratio statistics from the Granger causality test for the *gthi* time series of the 18 bands, in the wet and dry periods. 'gth' is the band identifier, 'Codex' for species is as listed in S1 Appendix: Table B. The two independent variables were soil moisture potential (SMP) and logger temperature (TEMP), as used before in the GLS-arima models.

Number	Station	Tree	Codex	gth	Wet period		Dry period	
					SMP_0	$TEMP_0$	SMP_0	$TEMP_0$
1	1	1	Mw1	g11	30.06***	13.33***	3.86*	0.94^{ns}
2	1	2	Sf1	g12	9.04***	2.49^{o}	2.17^{ns}	1.32^{ns}
3	1	4	Lb1	g14	3.66*	2.20^{ns}	6.13**	3.95*
4	1	5	Sf2	g15	25.76***	6.67***	14.77**	14.25***
5	2	2	Pm1	g22	7.94***	2.03^{ns}	5.45**	3.90*
6	2	3	Dm1	g23	8.03***	5.61**	8.37***	7.91**
7	2	4	Mw2	g24	5.10**	1.49^{ns}	6.86**	4.05*
8	2	5	Sf3	g25	25.43***	11.16***	5.41**	3.70*
9	3	1	Mw3	g31	15.85***	12.05***	7.47**	4.58*
10	3	2	Sp1	g32	0.10^{ns}	1.06^{ns}	16.38***	10.54***
11	3	3	Lb2	g33	8.30***	0.18^{ns}	4.79*	4.11*
12	3	4	Dm2	g34	9.93***	1.98^{ns}	8.13***	1.76^{ns}
13	3	5	Dm3	g35	10.40***	0.50^{ns}	4.00*	5.80**
14	4	1	Sf4	g41	2.52^{o}	1.95^{ns}	--	--
15	4	2	Dm4	g42	24.42***	13.43***	--	--
16	4	3	Pm2	g43	15.35***	2.37^{o}	--	--
17	4	4	Sp2	g44	27.89***	2.77^{o}	--	--
18	4	5	Pm3	g45	15.12***	2.25^{ns}	--	--

***, $P \leq 0.001$; **, $P \leq 0.01$; *, $P \leq 0.05$; ^{o}, $P \leq 0.10$, ^{ns}, $P > 0.10$.

S1 Appendix: Table E. Linear regression statistics of SMP and TEMP estimates from GLS-arima regressions on relative maximum diurnal change in stem girth (*gthch*$_{max}$); of the trees with bands (β_0, intercept; $\beta_1$slope) in the dry period (g15 excluded, n = 12), for (a) single-term models with the three lags of no and one or two days, and (b) two-term models with lags of no and one day crossed. These regressions were applied to predict the missing estimates for station 4 in the dry period.

(a) Single-term models

variate	lag	β_0	β_1	F-value[a]	P(F)	R^2(%)[b]
SMP	0	0.593	–0.302	27.2	< 0.001	62.6
	1	0.604	–0.279	23.0	< 0.001	58.2
	2	0.546	–0.232	24.8	< 0.001	58.8
TEMP	0	–0.523	0.686	9.1	0.013	25.2
	1	–1.128	0.490	9.0	0.013	21.9
	2	–1.420	0.876	35.1	< 0.001	67.5

[a] df = 1, 10, [b] adjusted.

(b) Two-term models

variate	SMP-lag	TEMP-lag	β_0	β_1	F-value[a]	P(F)	R^2(%)[b]
SMP	0	0	0.582	–0.259	17.73	0.002	60.3
	0	1	0.498	–0.284	37.17	< 0.001	76.7
	1	0	0.602	–0.241	16.19	0.002	58.0
	1	1	0.530	–0.252	27.13	< 0.001	70.4
TEMP	0	0	–0.095	0.411	4.68	0.056	25.1
	0	1	–0.604	0.136	0.86	0.375	–1.3
	1	0	–0.316	0.511	7.20	0.023	36.1
	1	1	–0.629	0.261	4.99	0.050	26.6

[a] df = 1, 10, [b] adjusted.

S1 Appendix: Fig A. Relationship between soil moisture potential (SMP, sqrt-transformed) and the 20-day running rainfall total in the wet and dry periods. The fitted curves were: wet, $Y = -5.61 - 8.80\cdot(0.9824^{X}) + 0.00439\cdot X$ (R^2= 34.3%, n = 550); dry, $Y = -1.30 - 30.9\cdot(0.9837^{X}) - 0.0270\cdot X$ (R^2= 68.4%, n = 98).

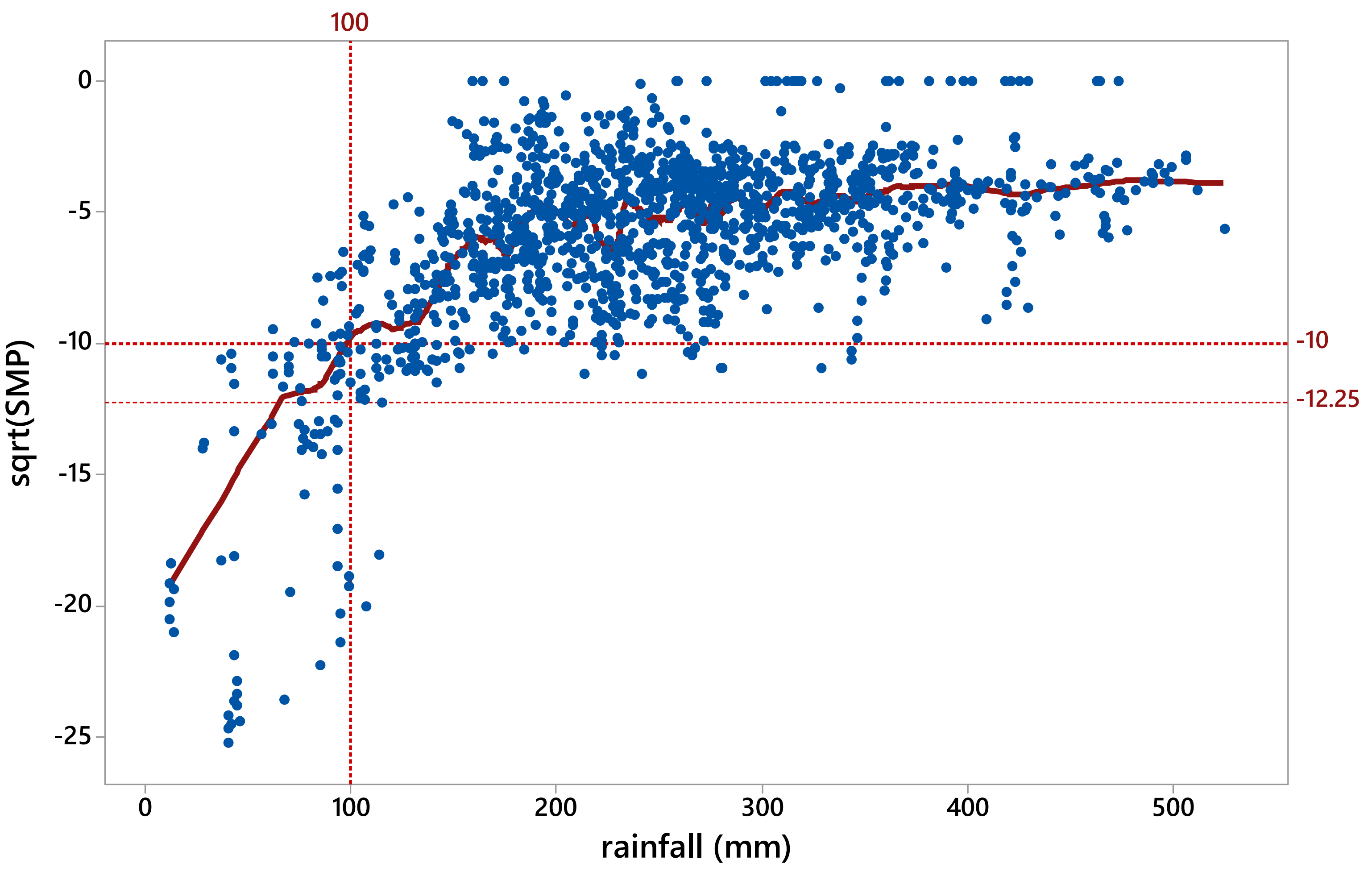

S1 Appendix: Fig B. Non-linear regression model fits of the relationship between square-root transformed soil moisture potential (SMP_sqrt) and the 20-day rainfall totals (rft), for the wet and dry periods at Danum.

WET: SMP_sqrt = −5.611 − 8.80*(0.9824^rft) + 0.00439*rft; F = 96.7, df = 3, 546, P < 0.001.

DRY: SMP_sqrt = −1.30 – 30.9*(0.9837^rft) − 0.0270*rft; F = 71.0, df = 3, 94, P < 0.001.

Fig. S2a

WET period

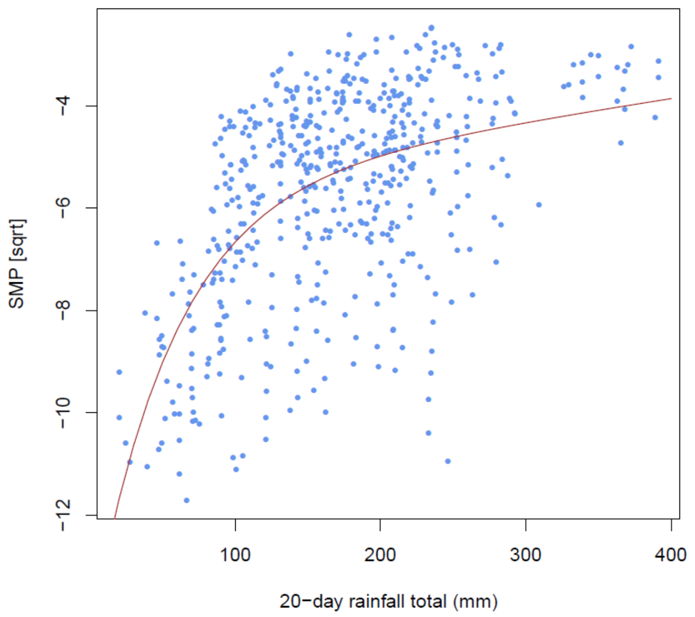

Fig. S2b

DRY period

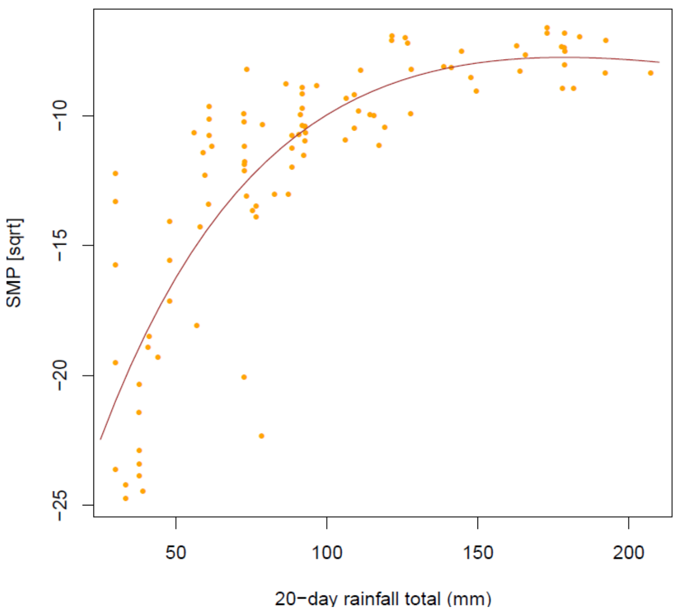

S1 Appendix: Fig C. Mean diurnal courses of (a) soil moisture potential (SMP), across all stations; and (b) logger temperature (TEMP), for station 1 as example, in the wet and dry periods. For SMP, means are for the 30 successive wet sub-periods (LL and UL – the 95% confidence limits), but for the three dry sub-periods separately.

(a) SMP

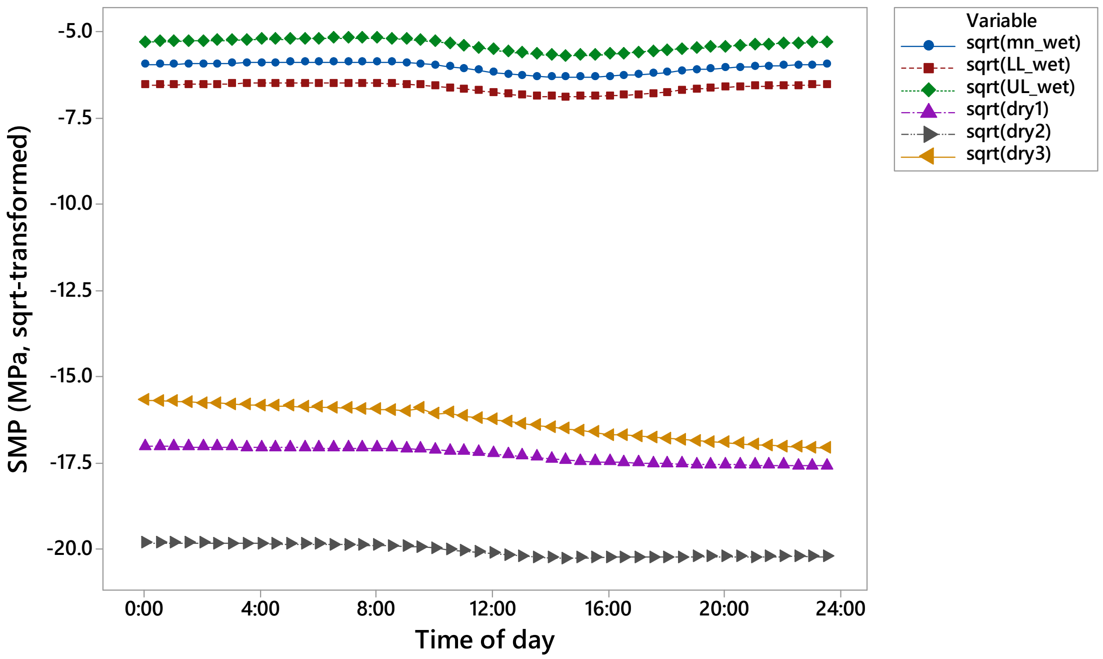

(b) TEMP

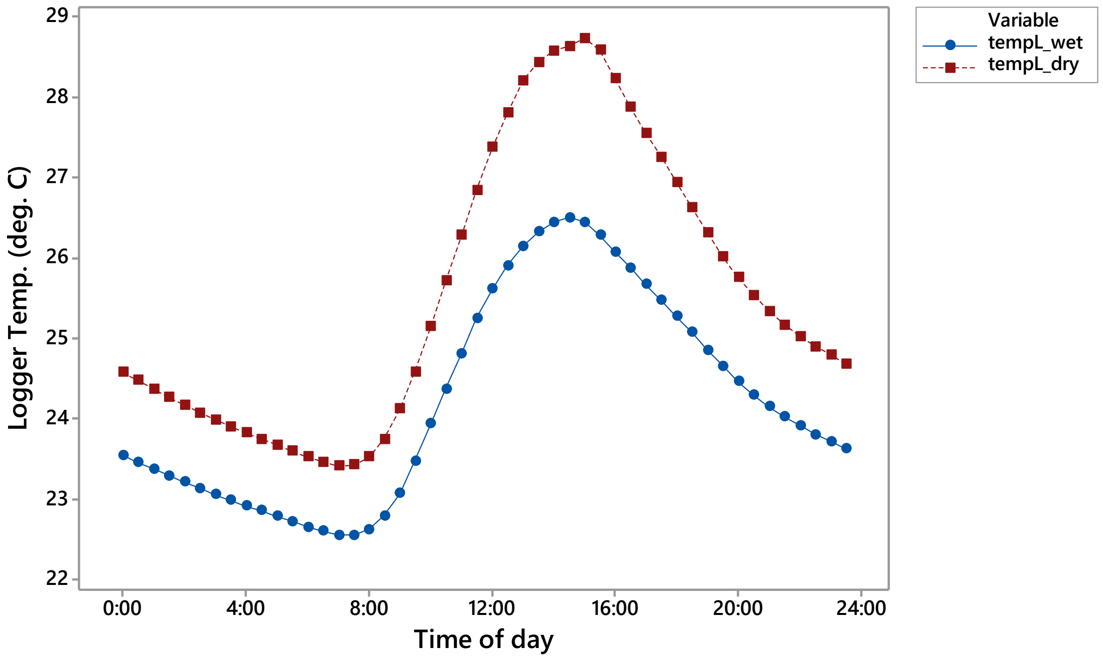

S1 Appendix: Fig D. Comparison of the SMP_{-1} estimates from GLS regression models with or without 'rain' (20-d-rft, sqrt-transformed) as an addition term; for (a) the wet (excluding the four bands where GARCH models were required (see main text), and (b) the dry, periods. Excluding the strong and unusually behaving outlier (g12) in the wet period, and the two bands with atypical 1-day lagged rainfall dependencies in the dry one, corresponding non-linear curve fits are shown in (c) and (d).

(a) Wet

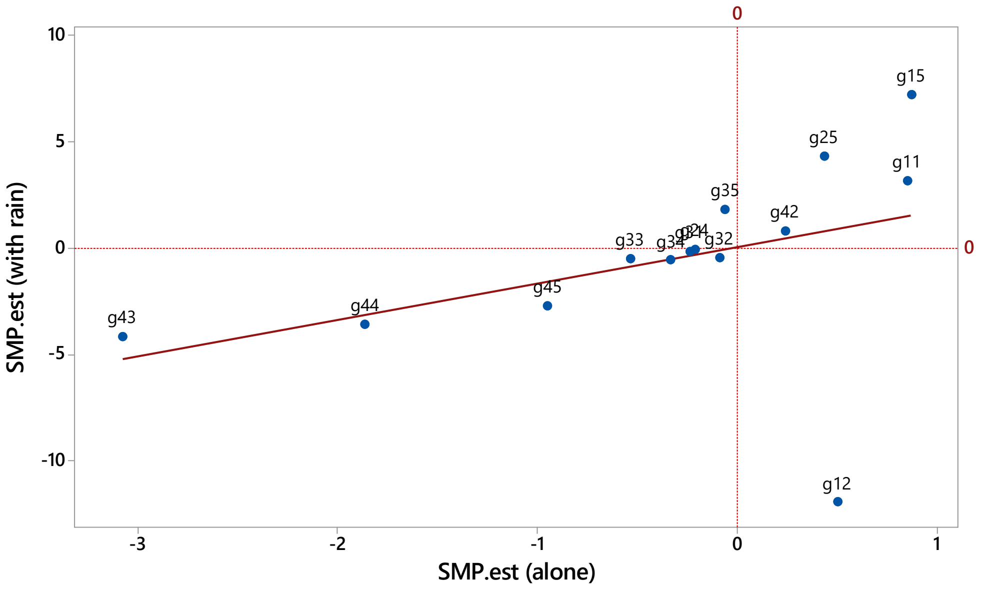

(b) Dry

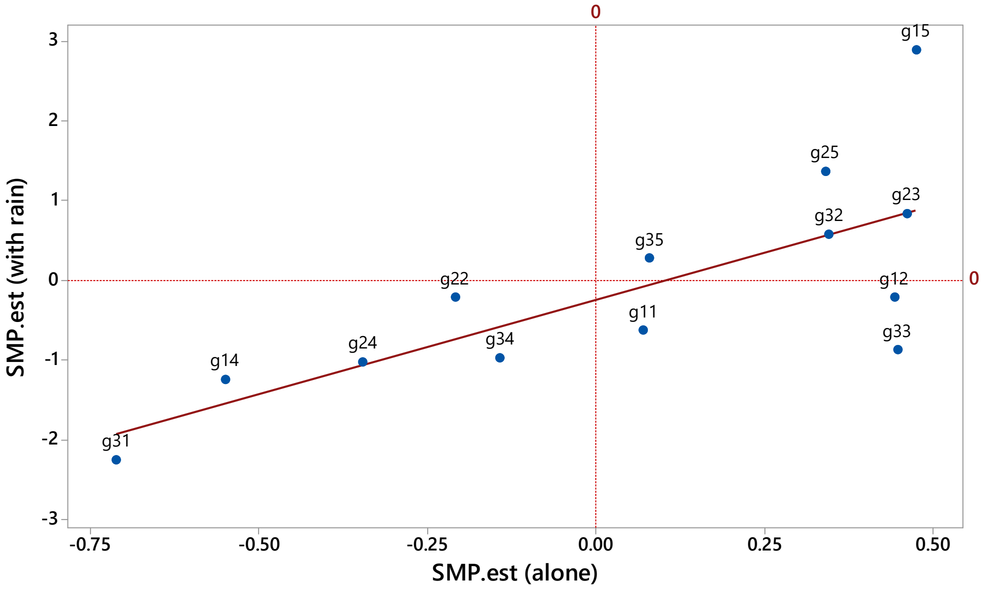

(c) Wet - fitted curve
[Y = 0.9429 + 4.286 X + 0.8654 X^2; R^2adj = 85.9%]

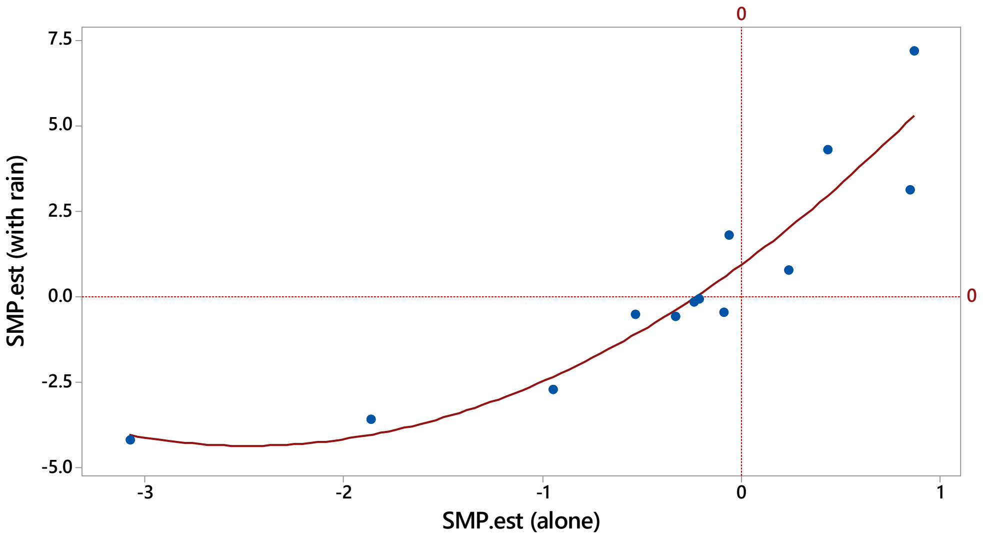

(d) Dry - fitted curve
[Y = - 0.2025 + 3.352 X + 1.442 X^2; R^2adj = 77.1%]

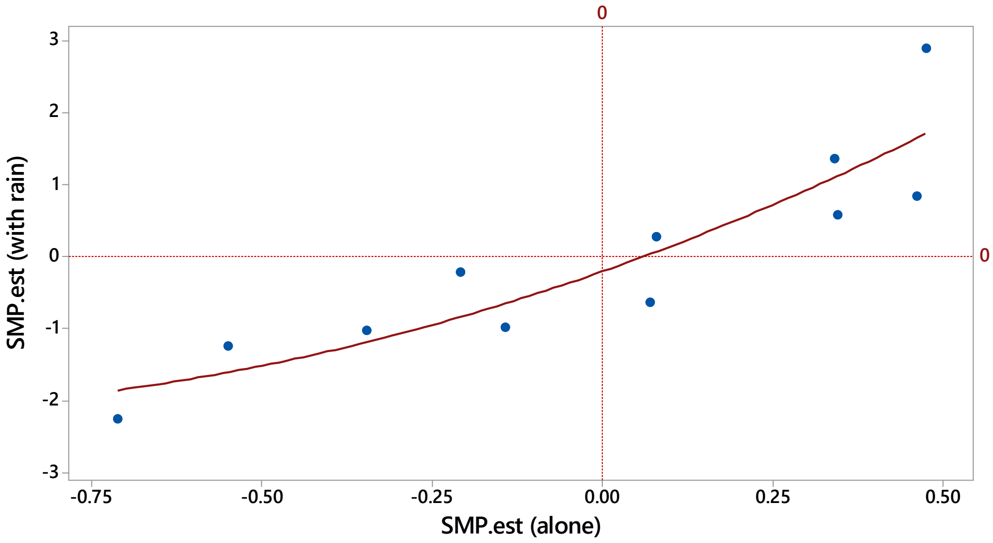

S1 Appendix: Fig E. Standardized SMP_{-1} versus $TEMP_0$ coefficients graphed together; for (a) wet and (b) dry periods for the 18 respective 13 trees analyzed for their gthi time-series, and their corresponding dry- and wet-period (c) SMP_{-1} and (d) $TEMP_0$ values against one another, annotated with the station numbers. Large trees (*scl* = 1), filled circles; small trees (*scl* = 2), filled triangles. This is equivalent to Fig 7 in the main text where species labels are shown. Station locations are shown in Fig 1 in the main text.

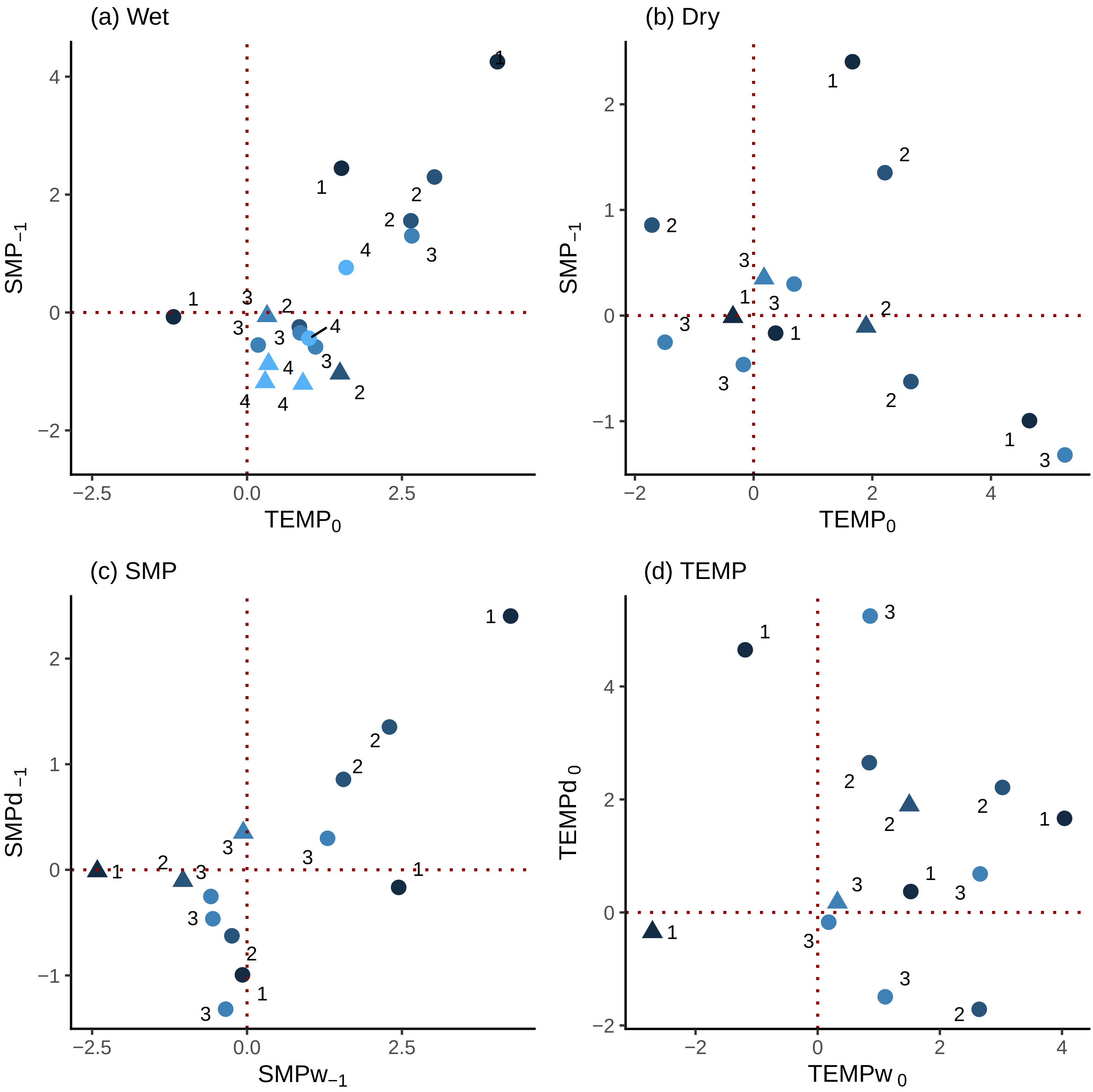

S1 Appendix: Fig F. Dependence of the SMP_{-1} and $TEMP_0$ estimates from the single-term GLS-arima regression models on the inverse-distance weighted basal area abundance (BA/d) of neighbouring trees to the banded one with a 5-m radius for the available small trees (*scl* = 1), in the wet and dry periods. Species codes are as shown in Table 2 of the main text (or in S1 Appendix: Table A).

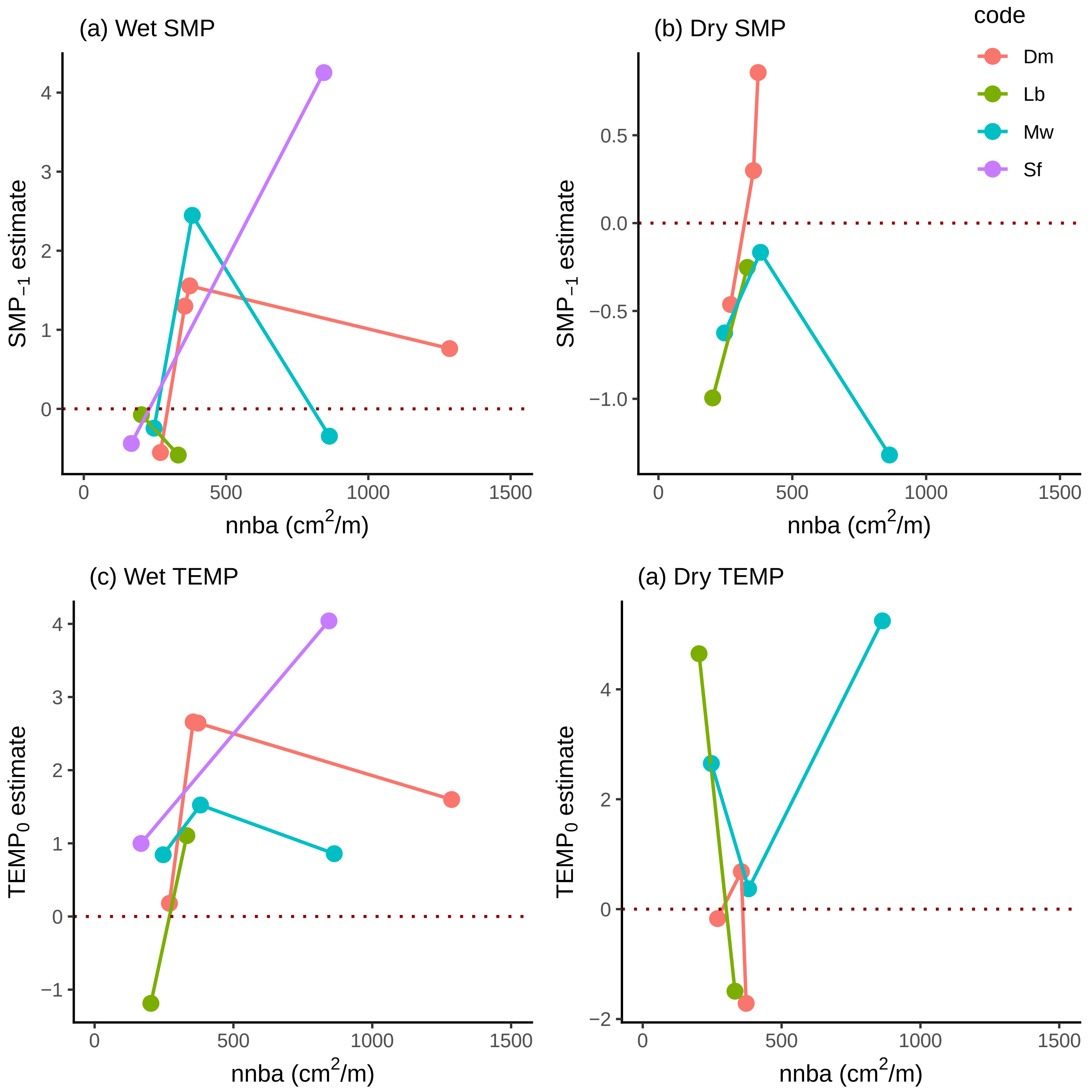

Supplement: S1 Appendix — (PDF) [file pone.0270140.s001.pdf]
