# Supplementary material for: Stem girth changes in response to soil water potential in lowland dipterocarp forest in Borneo: An individualistic time-series analysis
Source: PLoS One. 2022 Jun 30;17(6):e0270140. doi: 10.1371/journal.pone.0270140 (PMC9246238; doi:10.1371/journal.pone.0270140)
Supplement: S2 Appendix — (PDF) [file pone.0270140.s002.pdf]

S2 Appendix: Text 1. Further details of analysis of SMP data

Stations 1 and 2 were ‘LS’ (lower slope) and stations 3 and 4 ‘R’ (ridge) in the topographic classification (Fig. 1), and averaging these pairs’ SMP-values fortunately allowed almost complete runs of data over the 3 months, and afforded a useful comparison with Gibbons and Newbery (20). That study recorded SMP at ‘LS’ and ‘R’ locations within the same plots (slightly different sample layout) and built models to predict it from daily rainfall. SMP had then been measured with calibrated glass psychrometers. These last instruments are more reliable than the equitensiometers used in the present study, especially for lower potentials. The authors found a weighted rainfall index per day defined as $w_{40} = {}_{n=1}\Sigma^{40} (r_n/n)$, i.e. the sum of the preceding 40 days’ rain and each weighted by its day number (e.g. $n = 1$ for the day before). This led to two equations (w_{40} in cm; SMP in MPa): one for ‘LS’, SMP = −0.0096 − $1.4070b^{w40}$; the other for ‘R’, SMP = −0.0525 − $2.0280b^{w40}$, with b = 0.7554. The equations were applied to the complete rainfall series at Danum, and for this study between 01.03.2007 and 30.07.2011 to give ‘predicted’ SMP at the ‘LS’ and ‘R’ stations, and thereby match the (non-missing) observed equitensiometer values.

Comparisons between the two approaches to adjusting SMP-values and their comparison with predicted values led to two clear results. (1) For ‘LS’ and ‘R’ (averaging SMP-values across the two stations each), ‘offset’ and ‘strict’ SMP-values were closely and similarly related. SMP-values were first transformed as SMPtr = √│SMP│ (e.g. −150 kPa becomes 12.25), and allowing the lower (more negative) original values (now relatively more positive transformed ones) to weight accordingly against the many low and near-zero values, the linear regressions were $SMPtr_{LS}$ = 3.46 + 0.889 SMP_{LS} (R^2 = 91.5%, n = 1350) for ‘LS’, and $SMPtr_R$ = 3.27 + 0.890 SMP_R (R^2 = 90.4%, n = 1248) for ‘R’. Substituting an ‘offset’ threshold SMP = −150 kPa in these equations gives after back-transformation ‘strict’ thresholds of −97.9 and −101.8 kPa respectively. Hence, a threshold of −150 kPa with

‘offset’ translates to −100 kPa with a ‘strict’ setting. (2) Regressing ‘offset’ and ‘strict’ SMP-values against w_{40}-predicted SMP (days where the latter was ≤ − 50 kPa for ‘L’, ≤ − 100 kPa for ‘R’; *n* = 89 and 94 respectively) showed significant, though not particularly strong, quadratic relationships (R^2-values of 57.3 to 67.0%). ‘Offset’ SMP-values gave much closer to w_{40}-predicted values than ‘strict’ ones at ‘LS’ (the latter underestimating by c. 20%), whilst both ‘offset’ and ‘strict’ (more so) underestimated w_{40}-predicted values at ‘R’, by c. 30 and 45% respectively. It seems then that SMP-values on ridges were likely, in reality, to have been lower (taking the psychrometer values too as being the more reliable) than those recorded by the equitensiometers.

S2 Appendix: Text 2. Change in dendrometer band length due to temperature

The UMS Manual and Data Sheet for the D6 band (UMS, München, Germany) suggest a coefficient of < 1 µm/m/°K for the Invar steel cable and state a 'temperature error' of < 4 µm/°K' for the whole dendrometer. The expansion of the dendrometer is made of two components: (a) the cable itself (λ_1 = 1 µm/m/°K) and (b) the 'block-and-plate' (expansion value unstated by UMS), which together give the < 4 µm/m/°K overall. Since the cables (bands) are of different lengths on the various trees, a correction for each tree's band can be found for them independently. The 'block-and-plate' (which is the same construction for each band) should, by deduction, account for a < 4 - < 1 = ~ 3 µm/m/°K difference (λ_2).

Daily temperature differences ($\Delta T = T_{t+1} - T_t$) were found for each tree's band, corresponding to the 1-day increments in *gbh*. When ΔT was positive the dendrometer would have expanded and therefore adjusted the *gthi* should be smaller, and when the ΔT was negative the converse the case. The adjustment was: $gthi_{adj} = gthi - \{((mgbh/100) \cdot \lambda_1 \cdot \Delta T) + (\lambda_2 \cdot \Delta T)\}/k$, where k = 10 is a rescaling factor to convert the adjustment term in µm to cm·10^{-3} (m·10^{-5}), the unit for *gthi*. GLS regressions (p = 2, q = 2) were rerun using $ghti_{adj}$, for SMP with no lag, and separately for logger-TEMP with no lag, for the trees/bands in the wet and dry periods. Omitting the four trees where volatility was shown, and two very outlying, low, SMP- (slopes < 1.0) and two logger-TEMP-values (slopes < 1.5), regressions for the *n* = 25 species' adjusted versus unadjusted slope-values (estimates for wet {14} and dry {11} periods together) were very strong (R^2 = 99.8 and 99.4% respectively, and these two regression slopes very close to unity {0.978 and 1.015} with intercepts very near zero). The two outliers were also close to the lines for the 25 points but afforded high leverages).

Could diurnal changes in the bands have been in part due to daily temperature change? From the DVMC climate station, dry-bulb temperatures at 08:00 and 14:00 h in the wet period were 24.3 and 30.0 °C on average, and in the dry period correspondingly 25.3 and

31.7 ºC; ΔT being then 5.7 and 6.4 ºC for wet and dry periods. Although there are no records for temperatures at DVMC at midnight, Appendix 1: Fig. S4 shows for example that minimums were usually around 08:00 h. However, the *gthch* variable was calculated with 00:00 h as reference, and the mean logger temperatures at this time (t = 1) and the maximum at station 1 were 23.5 and 26.5 ºC in the wet, and 24.6 and 28.7 ºC in the dry, period; ΔT being 3.0 and 4.1 ºC for the two periods. Logger temperatures at 00:00 h were 1.0 to 1.1 ºC higher that those at 08:00 h. In the daytime the temperature sensor would, furthermore, have been somewhat insulated, from especially the higher temperature extremes, within the logger housing. The temperature curves for the other three stations were very similar. Taken together an average upper maximum diurnal ΔT experienced by the bands can be estimated at ~5.5 º C. Applying this in the formula for band expansion given above, a tree of 30 cm *gbh* would have maximally expanded 0.62 *gthch* units, and one of 100 cm *gbh* by 0.22 (the change being relative to *gbh* at t_1; most of the expansion in the 'block-and-plate'). These are very small values when judged against diurnal band fluctuations. Temperature change therefore affected the species' diurnal *gthch* patterns very minimally, at least as far as the bands themselves were concerned.
