# Supplementary material for: Stem girth changes in response to soil water potential in lowland dipterocarp forest in Borneo: An individualistic time-series analysis
Source: PLoS One. 2022 Jun 30;17(6):e0270140. doi: 10.1371/journal.pone.0270140 (PMC9246238; doi:10.1371/journal.pone.0270140)

S3 Appendix: Table A. Statistics for the days within the studied wet and dry periods; for which records of dry-bulb temperature and relative humidity at 14::00 h (Tdry14, RH14) were available at the Danum Valley Field Centre.

	n days	mean ± SD	median	minimum	maximum
Wet					
Tdry14 (°C)	357	30.0 ± 2.1	30.4	24.6	34.6
RH14 (%)	352	80.2 ± 8.4	79.7	61.3	98.3
Dry					
Tdry14 (°C)	67	31.4 ± 2.5	32.2	25.1	34.9
RH14 (%)	69	76.7 ± 10.8	75.0	59.5	100.0

S3 Appendix: Table B. Tables of estimates (± SE), with *t*-values and their probability levels, for GLS-arima regressions of daily stem girth increment on relative humidity recorded at 14:00 (RH14). (a) Coefficient of single fitted term 'RH14 residuals', taken from first regressions of RH14 on dry-bulb temperature at 14:00 (Tdry14), and (b) Coefficients of the interaction term in the nested model 'Tdry14 + Tdry14·RH14'. RH14 and Tdry14 were recorded at the DVFC climate station.

			(a) Residual RH term from one-term RH model				(b) Interaction term from two-term TEMP and RH model			
season	gth	spec	est	se	t(est)	P(t)	est	se	t(est)	P(t)
w	g14	Lb	0.4332	0.2694	1.068	0.109	0.01533	0.00852	1.799	0.073
d	g14	Lb	-0.6477	0.2652	-2.442	0.017	-0.02032	0.00769	-2.641	0.010

S3 Appendix: Fig A. Girth increment, *gthi*, of each tree in the wet period plotted against relative humidity (RH14, %); limited to three, thin temperature (Tdry14, °C) ‘slices’ — for those days on which the DVFC climate station did have recordings of both variables.

(a) Slice 1 (29.3 – 29.9 deg.C)

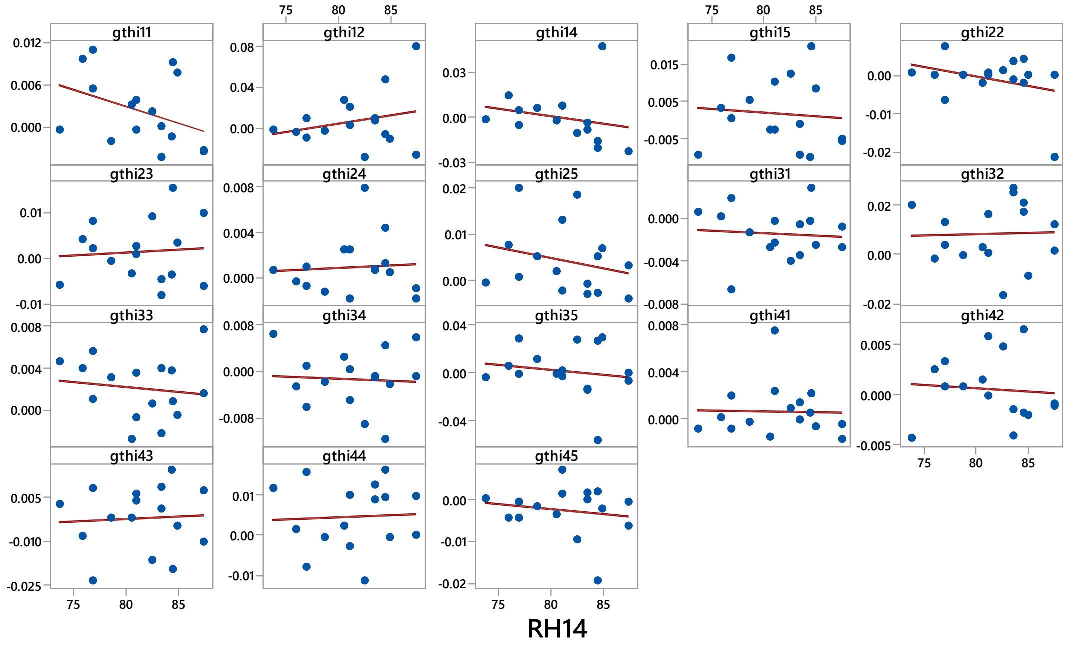

(b) Slice 2 (30.0 – 30.6 deg.C)

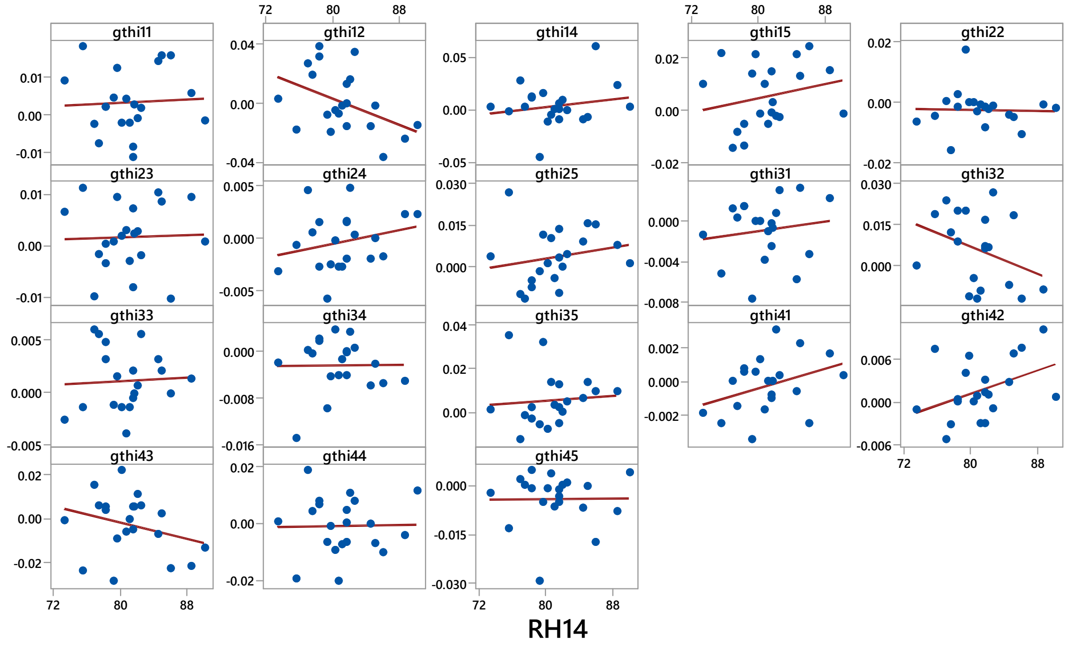

(c) Slice 3 (30.7 – 31.3 deg.C)

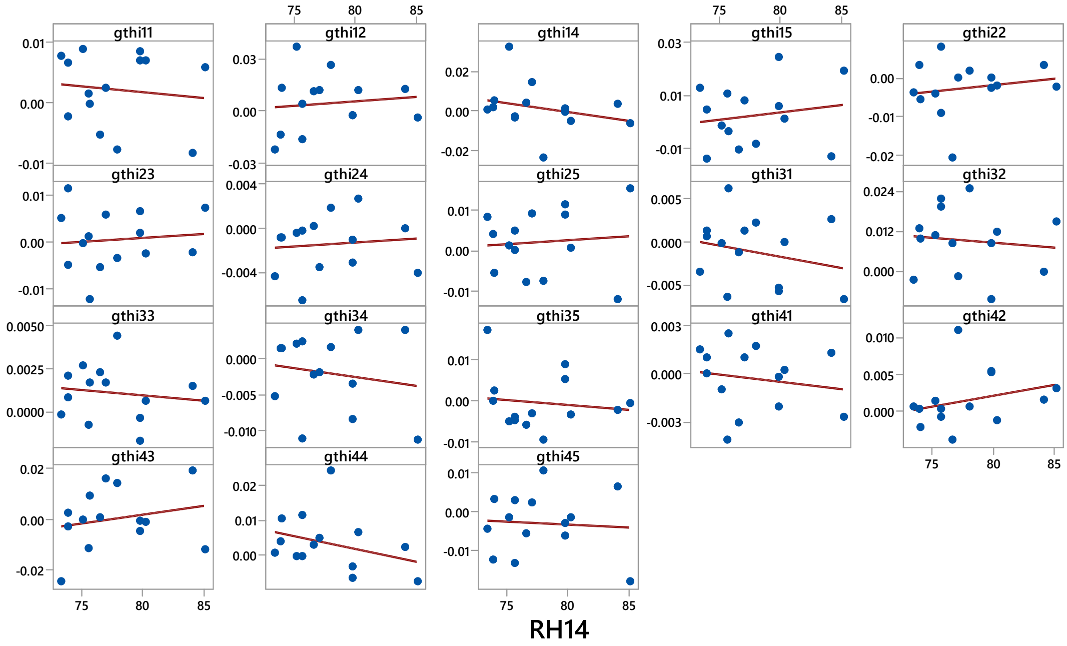

Supplement: S3 Appendix — (PDF) [file pone.0270140.s003.pdf]
