# Supplementary material for: Stem girth changes in response to soil water potential in lowland dipterocarp forest in Borneo: An individualistic time-series analysis
Source: PLoS One. 2022 Jun 30;17(6):e0270140. doi: 10.1371/journal.pone.0270140 (PMC9246238; doi:10.1371/journal.pone.0270140)
Supplement: S4 Appendix — (PDF) [file pone.0270140.s004.pdf]

S4 Appendix: Table A. Statistics for the quadratic regression fits of daily girth increment (gthi) of dendrobands on 13 trees as a function of rainfall in the current, and in the previous, day (on ln-scale) in the dry period, for days with ≥ 1 mm in the day. Explanation of the series is found in the main text. Probability values ≤ 0.02 are shown in bold font. Bands where the relationship is significantly stronger for the previous than current day are indicated in red.

series	band	code	Gbh	Rain on current day			Rain on previous day		
			(cm)	$R^{2\|a}$	F^b	P(F)	$R^{2\|a}$	F^c	P(F)
A(B)	g11	Mw	31	0.332	15.65	**<0.001**	0.071	3.22	0.048
	g12	Sf	97	0.034	2.03	0.141	0.235	9.92	**<0.001**
	g14	Lb	31	0.170	7.05	**0.002**	–0.007	0.80	0.457
	g15	Sf	27	0.345	16.52	**<0.001**	0.003	1.09	0.344
B	g22	Pm	74	0.148	5.67	**0.006**	0.069	2.97	0.060
	g23	Dm	30	0.137	5.28	**0.008**	–0.030	0.24	0.790
	g24	Mw	34	0.308	13.02	**<0.001**	–0.013	0.65	0.527
	g25	Sf	33	0.014	1.37	0.263	–0.024	0.38	0.689
	g31	Mw	34	0.249	9.95	**<0.001**	–0.021	0.44	0.647
	g32	Sp	78	0.097	3.92	0.026	–0.022	0.43	0.653
	g33	Lb	39	–0.033	0.13	0.876	0.440	21.81	**<0.001**
	g34	Dm	39	0.460	23.97	**<0.001**	0.002	1.07	0.352
	g35	Dm	48	0.132	5.10	**0.009**	0.006	1.16	0.322

a, adjusted R^2-value; df: [b], A(B) 2, 57; B 2, 52. [c] , A(B) 2,56; B 2, 51. [The 1 df less for the lagged regression error term is because the day before the starts of the series had no rain.]

S4 Appendix: Fig A.

Daily girth increment (gthi) as a function of rainfall in the current day,'rain', (on ln-scale) in the dry period. The 13 bands are presented pairwise in rows: on the left all days (i.e. those with zero rain included) are shown with a 'lowess' regression fit, and on the right for those days receiving ≥ 1 mm rainfall with a quadratic regression fit.

S4 Appendix: Fig B.

Daily girth increment (gthi) as a function of rainfall in the day before, 'rain_1', (on ln-scale) in the dry period. The 13 bands are presented pairwise in rows: on the left all days (i.e. those with zero rain included) are shown with a 'lowess' regression fit, and on the right for those days receiving ≥ 1 mm rainfall with a quadratic regression fit.

Fig A

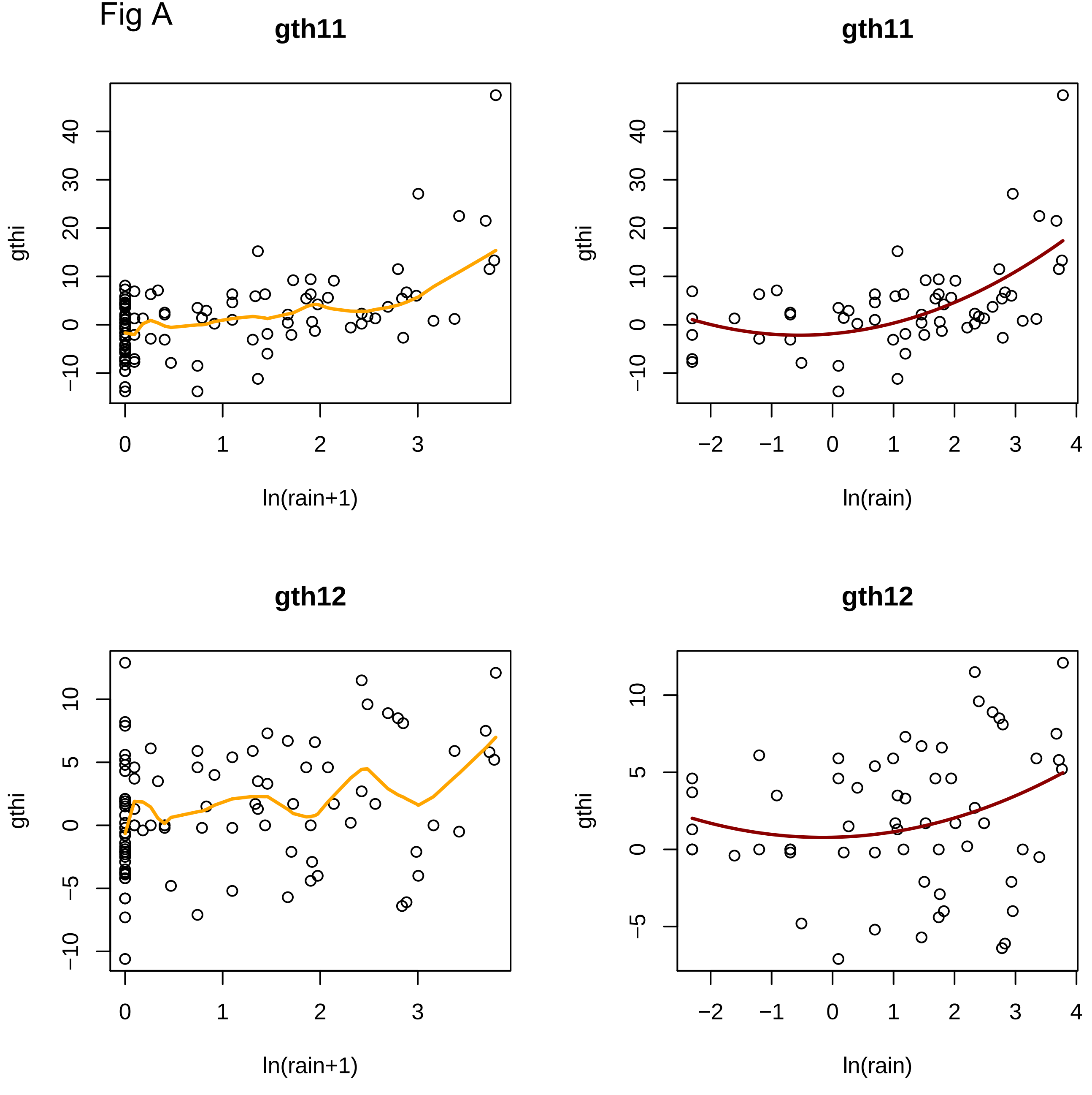
gth11
gthi
ln(rain+1)
gth11
gthi
ln(rain)
gth12
gthi
ln(rain+1)
gth12
gthi
ln(rain)

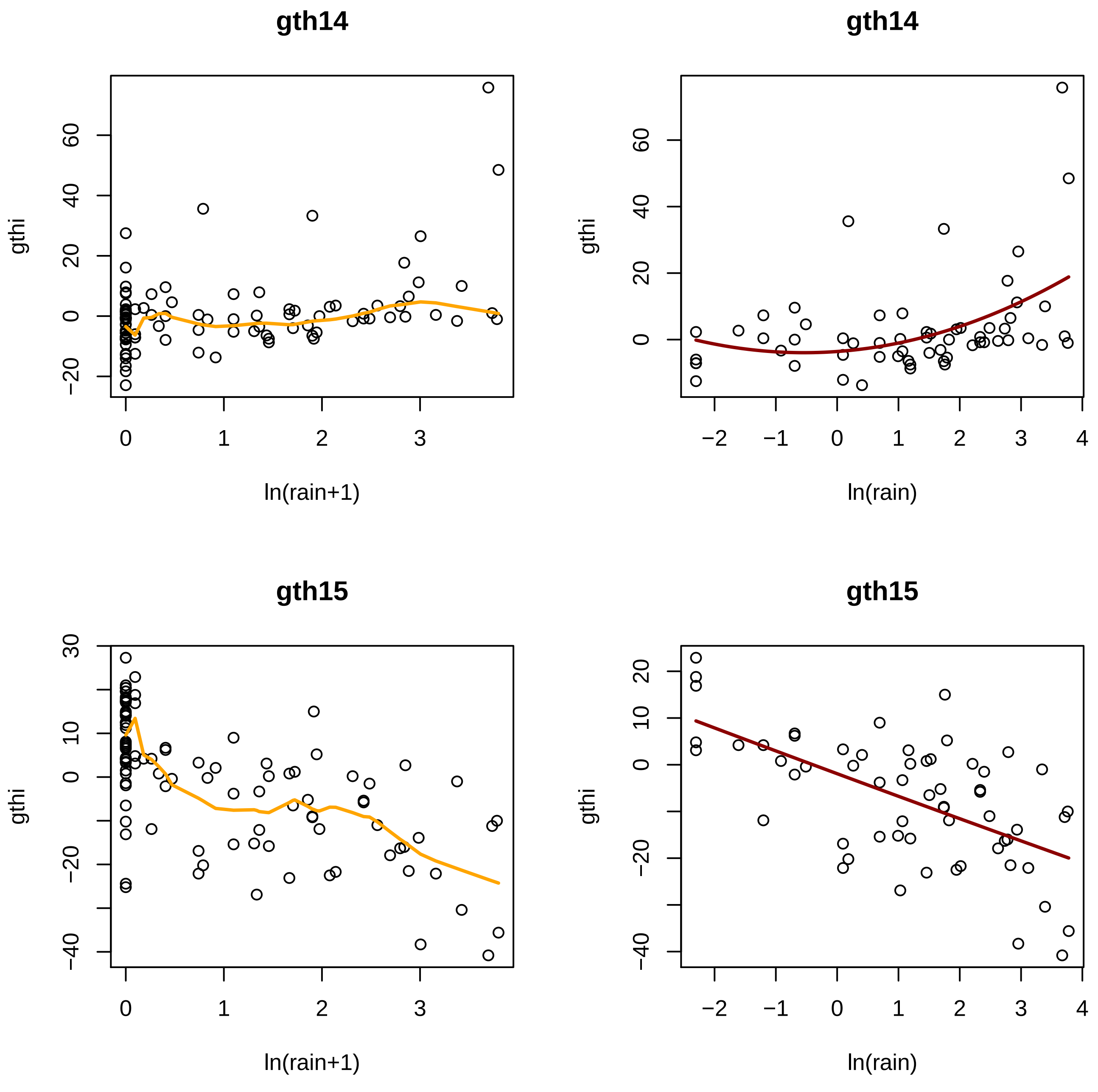
gth14
gth14
gth15
gth15
gthi
ln(rain+1)
ln(rain)
0
1
2
3
−2
−1
4
−20
20
40
60
−40
−20
10
30

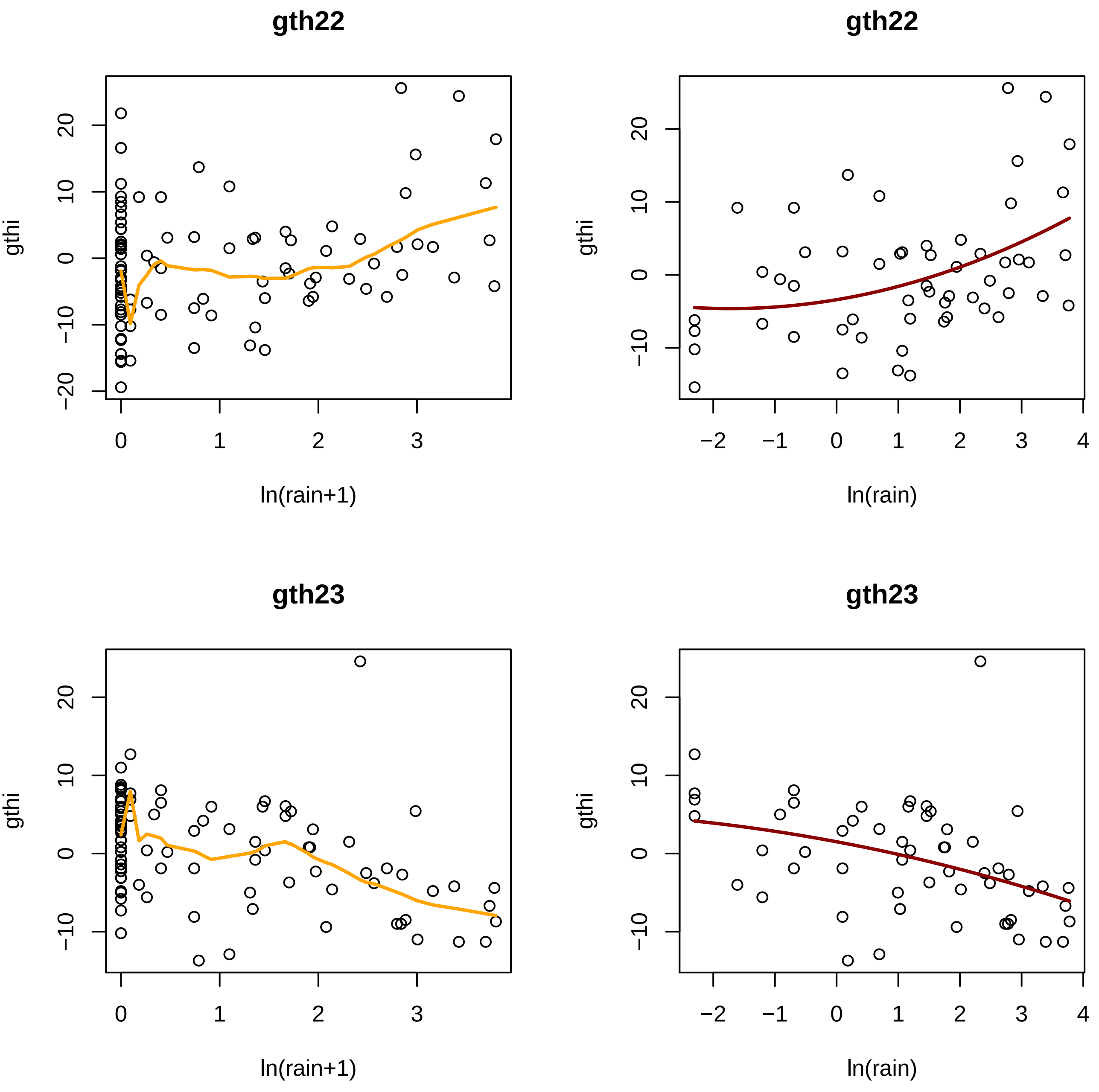

gth22
gthi
ln(rain+1)
−20
−10
0
10
20
0
1
2
3
gth22
gthi
ln(rain)
−10
0
10
20
−2
−1
0
1
2
3
4
gth23
gthi
ln(rain+1)
−10
0
10
20
0
1
2
3
gth23
gthi
ln(rain)
−10
0
10
20
−2
−1
0
1
2
3
4

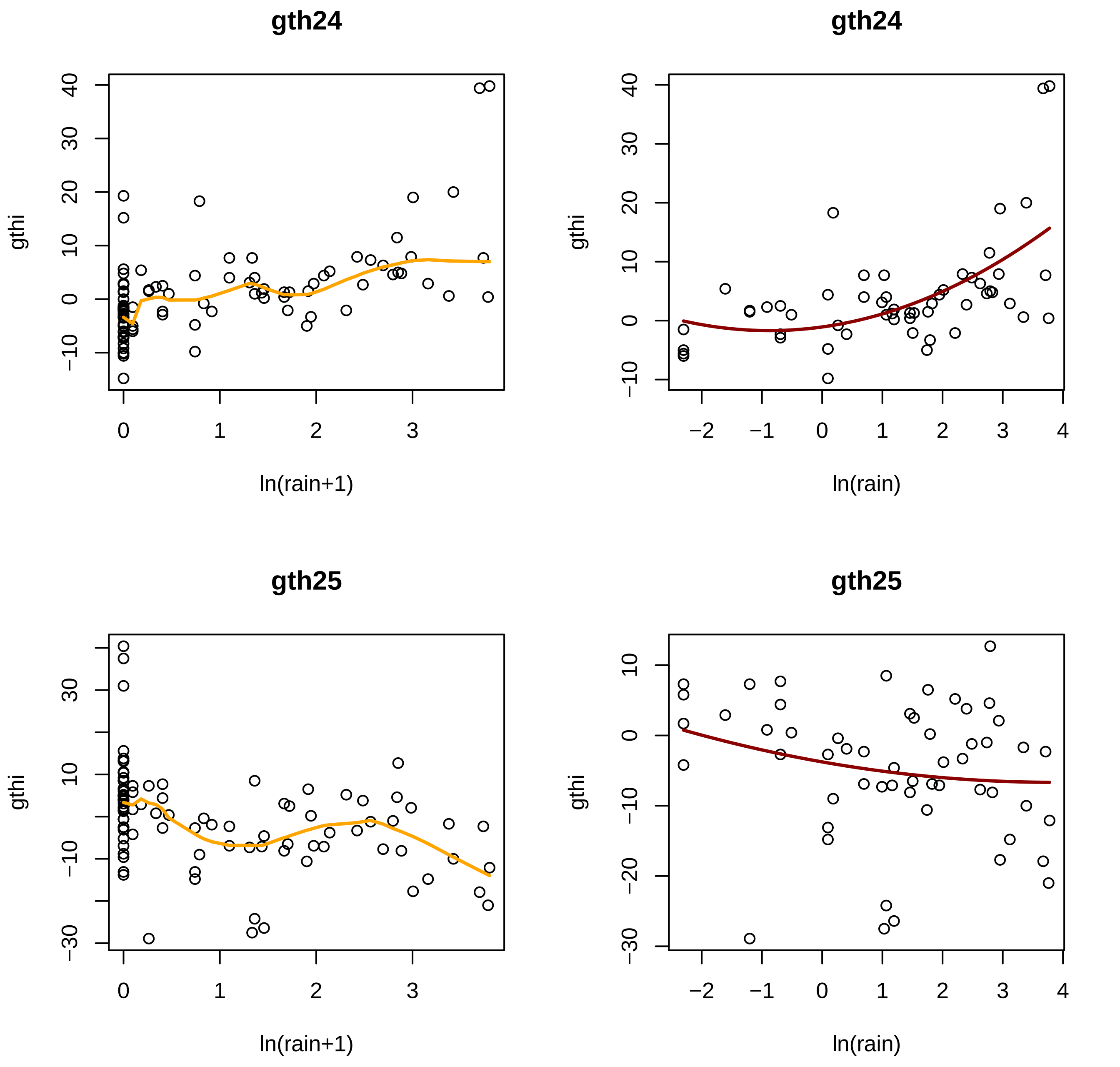
gth24
gthi
ln(rain+1)
gth24
gthi
ln(rain)
gth25
gthi
ln(rain+1)
gth25
gthi
ln(rain)

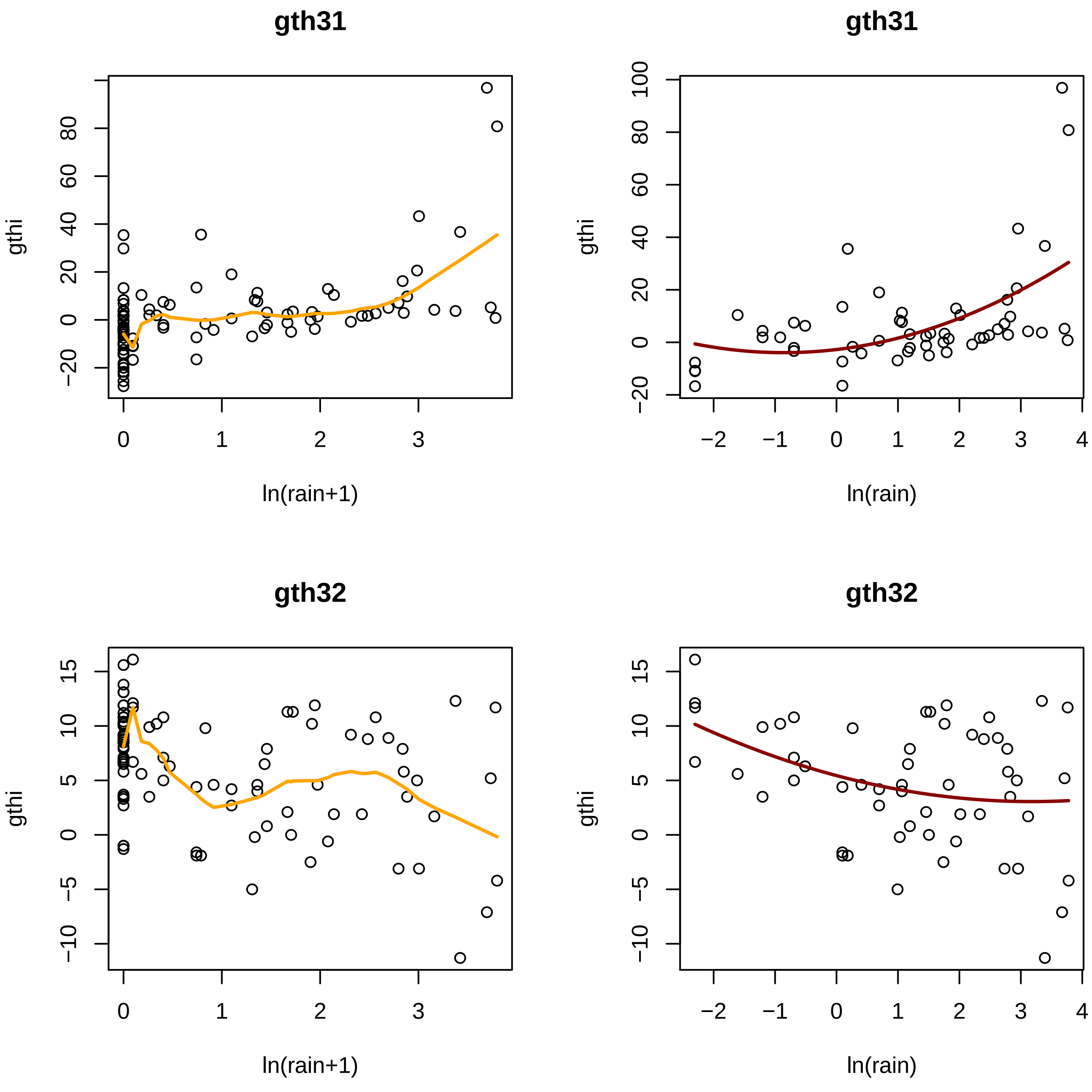
gth31
gthi
ln(rain+1)
gth31
gthi
ln(rain)
gth32
gthi
ln(rain+1)
gth32
gthi
ln(rain)

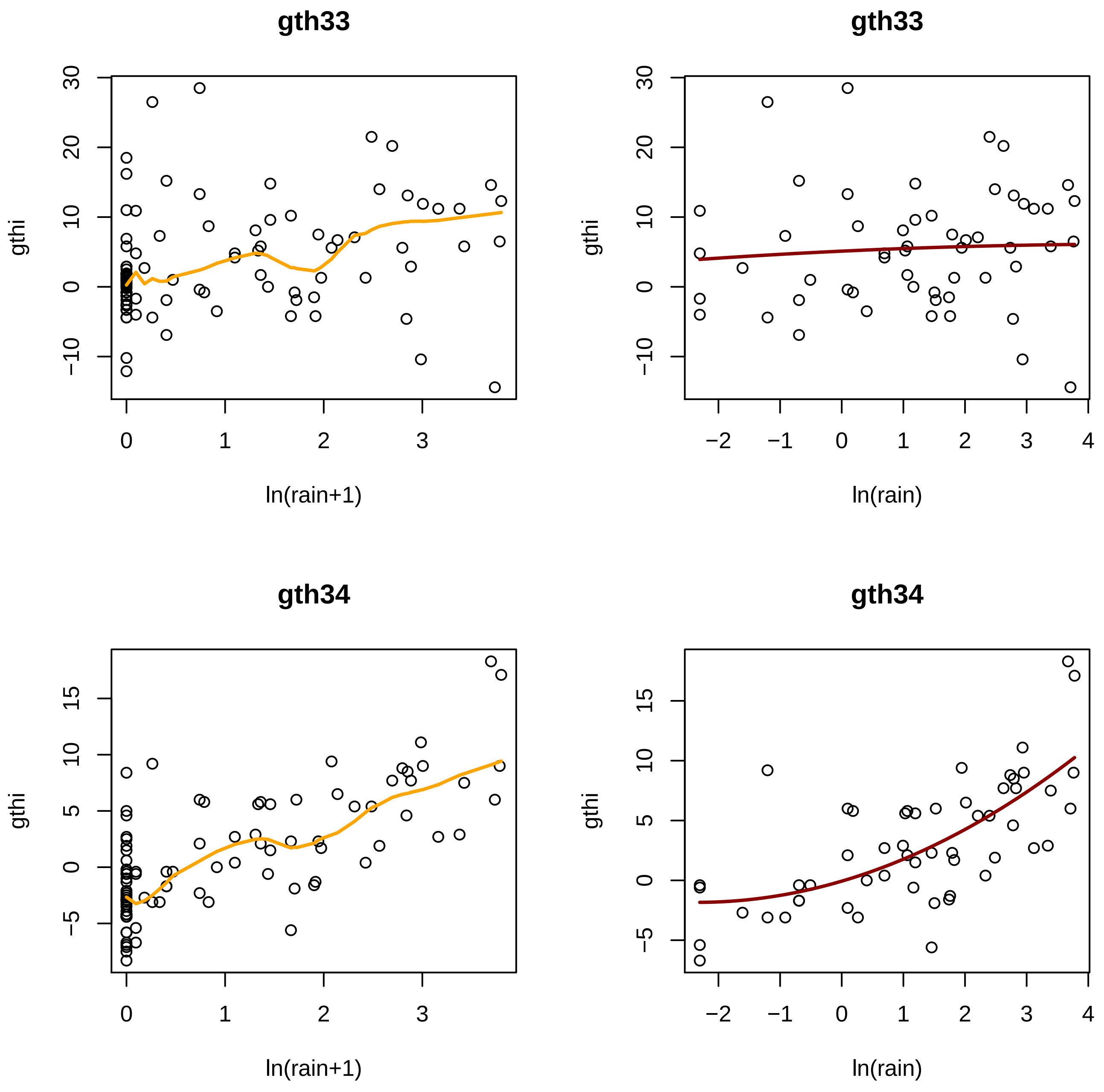
gth33
gth33
gth34
gth34
gthi
ln(rain+1)
ln(rain)
0
1
2
3
−2
−1
4
30
20
10
−10
15
5
−5

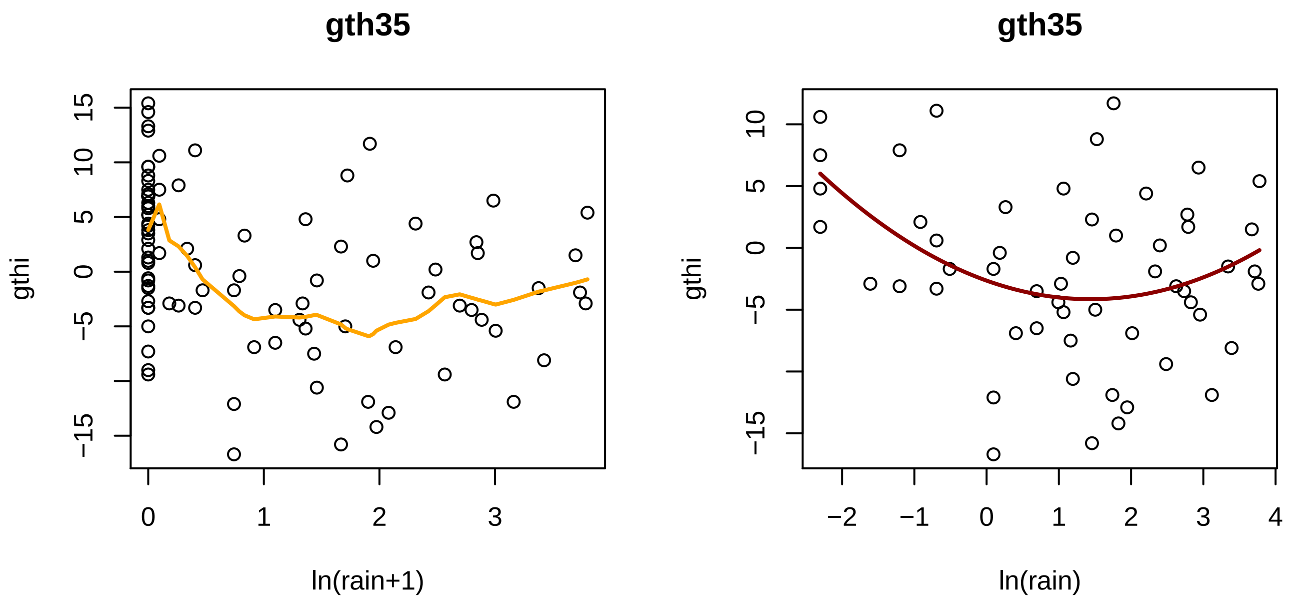
gth35
gthi
ln(rain+1)
gth35
gthi
ln(rain)

Fig B

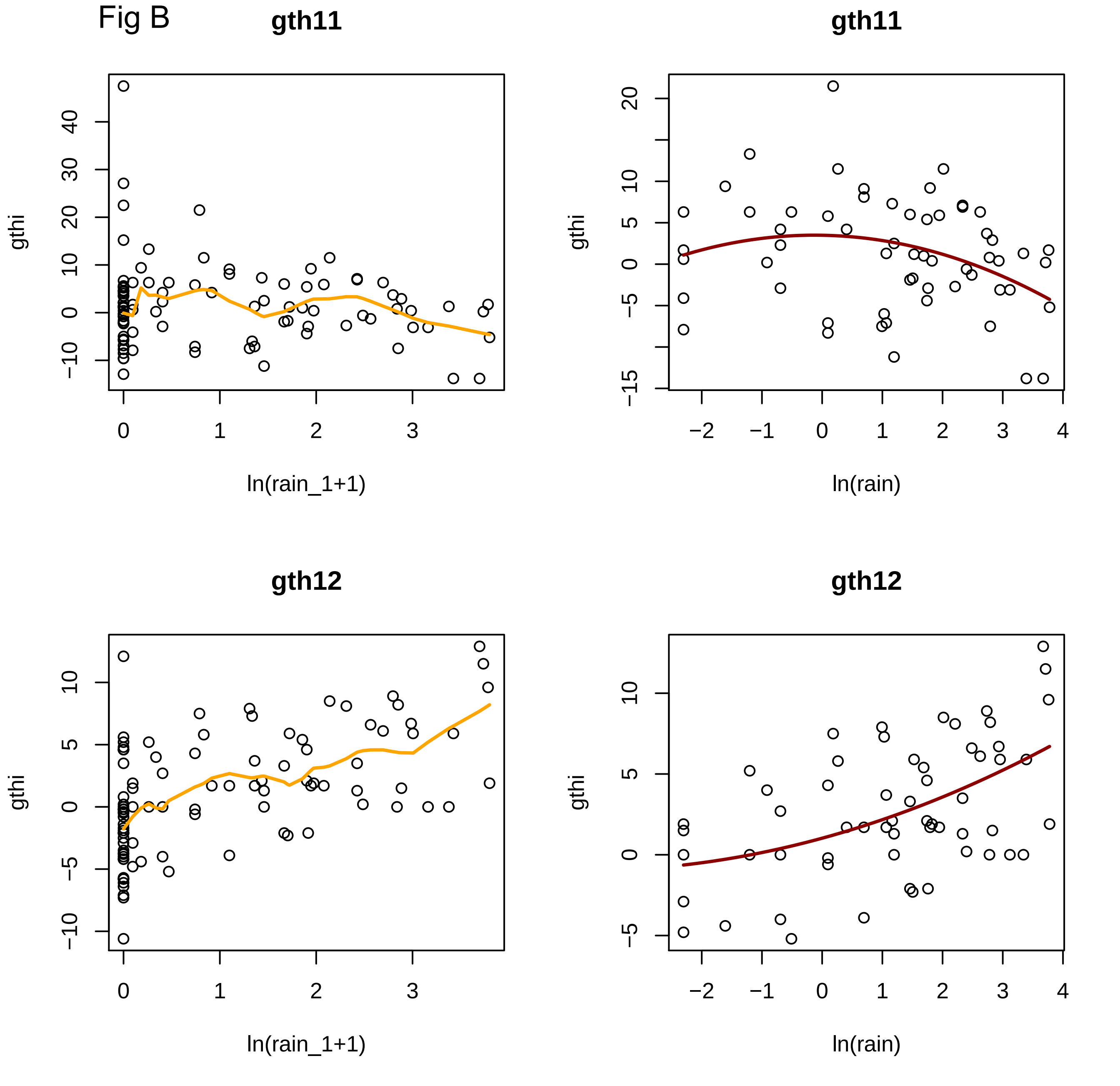
gth11
gthi
ln(rain_1+1)
gth11
gthi
ln(rain)
gth12
gthi
ln(rain_1+1)
gth12
gthi
ln(rain)

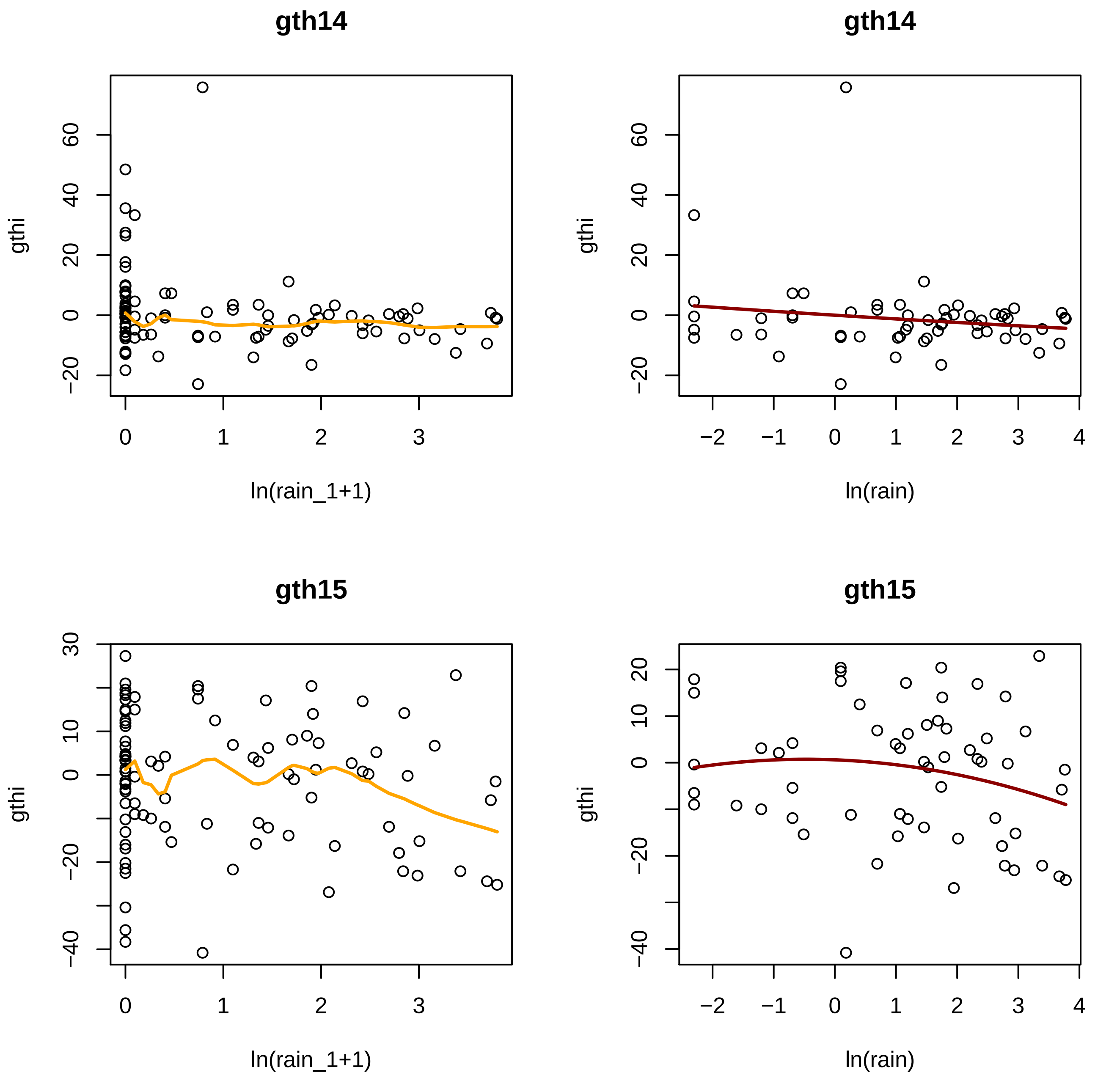

gth14
gthi
ln(rain_1+1)
gth14
gthi
ln(rain)
gth15
gthi
ln(rain_1+1)
gth15
gthi
ln(rain)

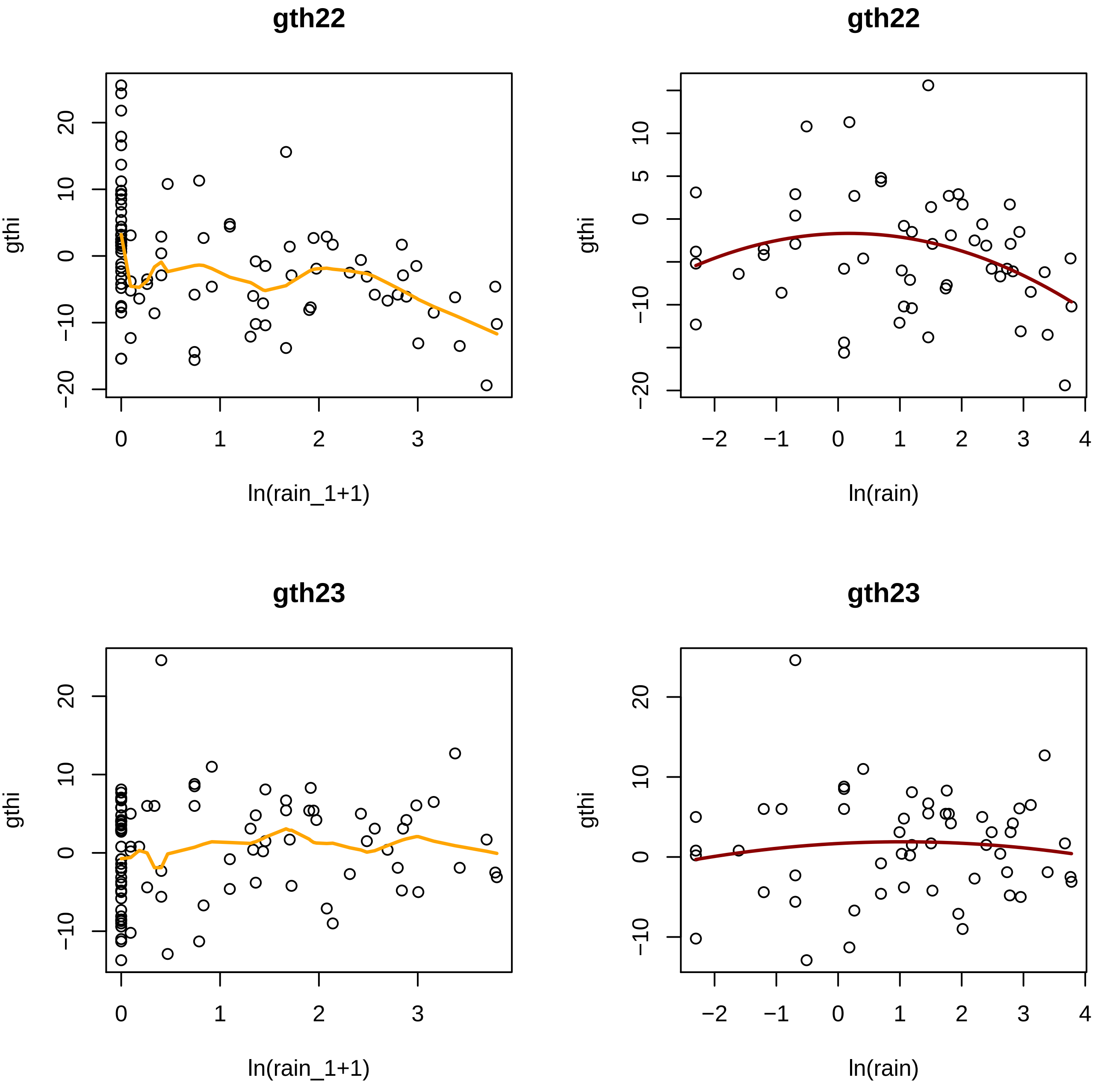
gth22
gth22
gth23
gth23
gthi
ln(rain_1+1)
ln(rain)
−20
−10
0
10
20
5
−2
−1
1
2
3
4

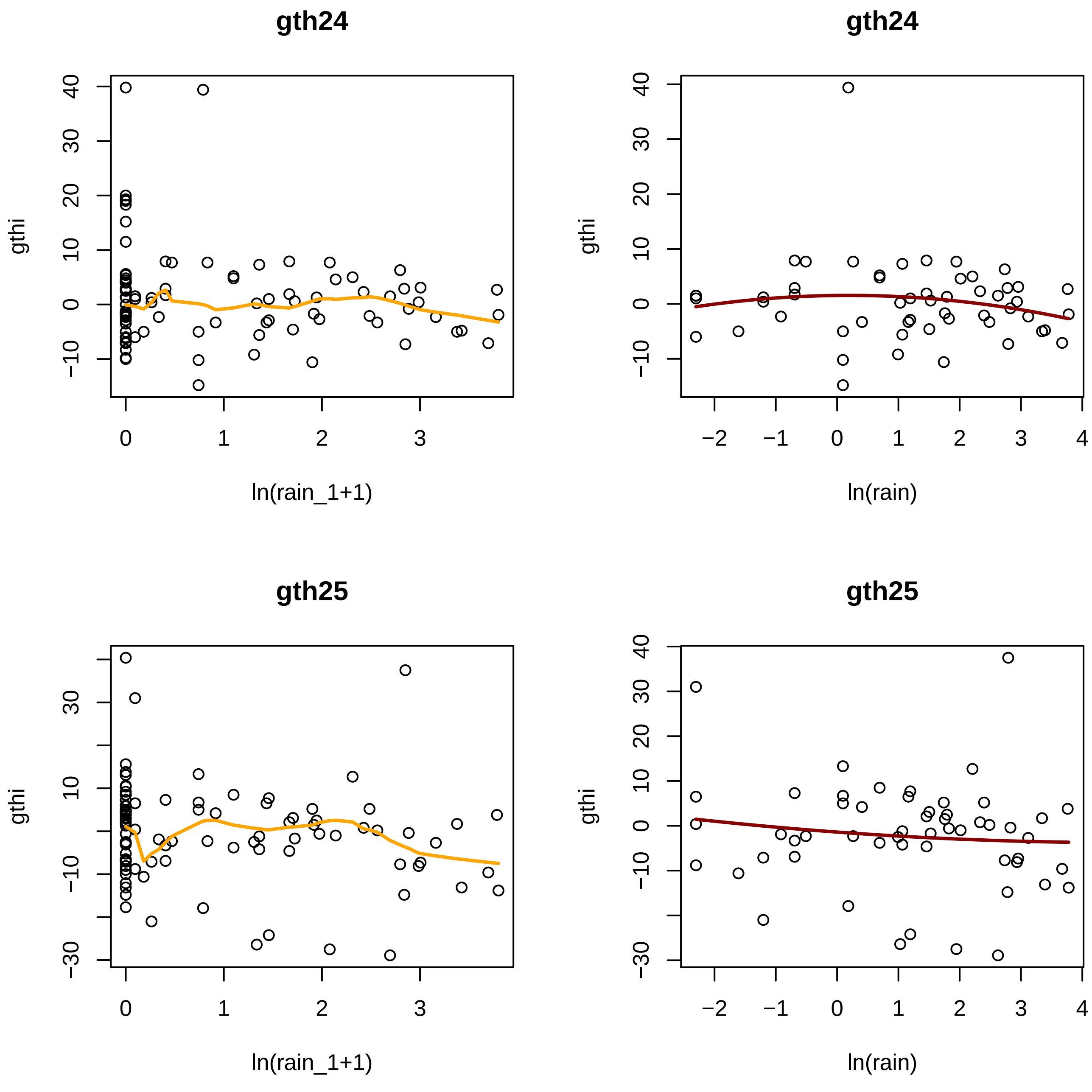
gth24
gthi
ln(rain_1+1)
gth24
gthi
ln(rain)
gth25
gthi
ln(rain_1+1)
gth25
gthi
ln(rain)
−10
0
10
20
30
40
−30
−2
−1
1
2
3
4

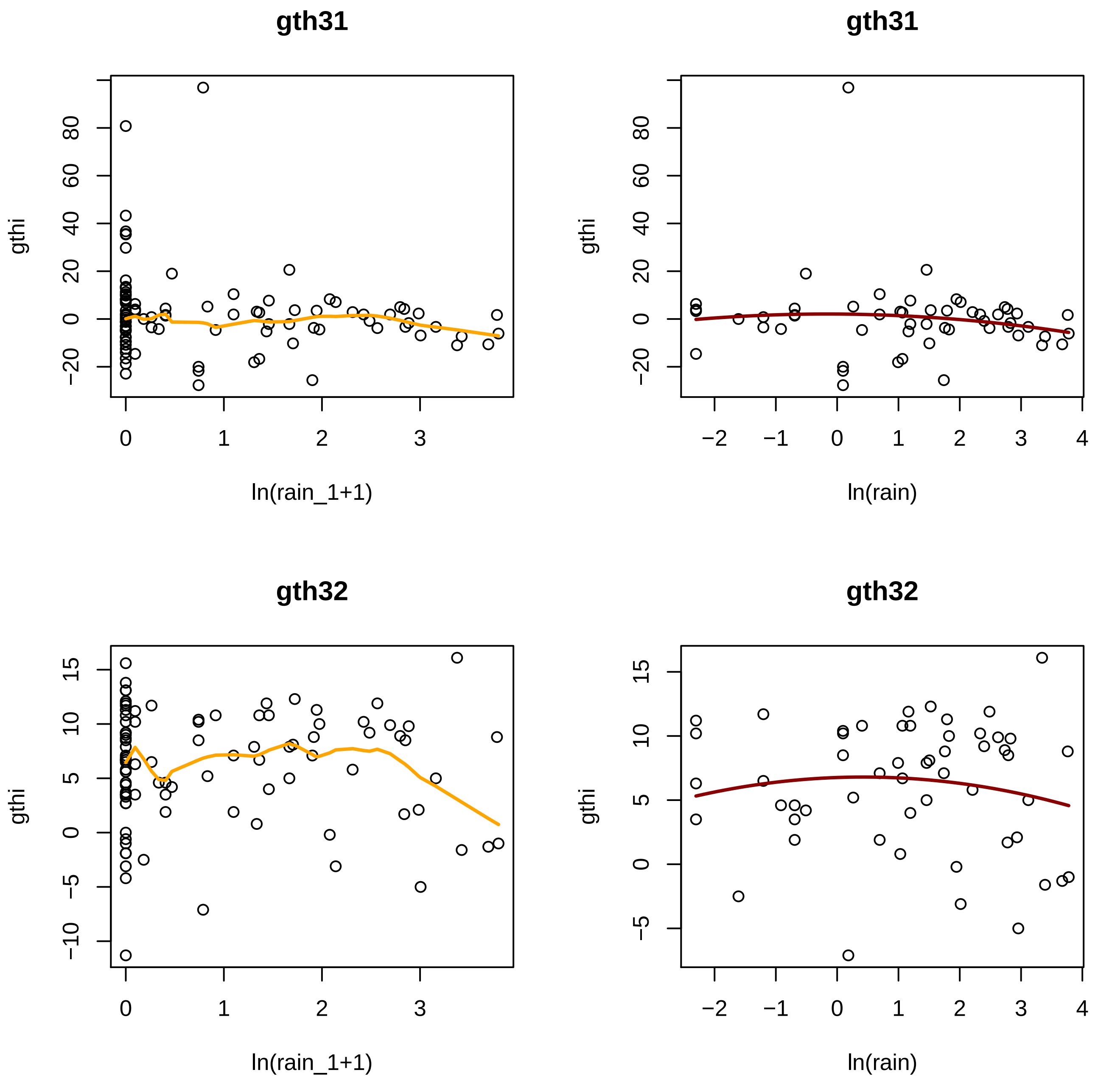
gth31
gthi
ln(rain_1+1)
gth31
gthi
ln(rain)
gth32
gthi
ln(rain_1+1)
gth32
gthi
ln(rain)
−20
0
20
40
60
80
−10
−5
0
5
10
15
0
1
2
3
−2
−1
4

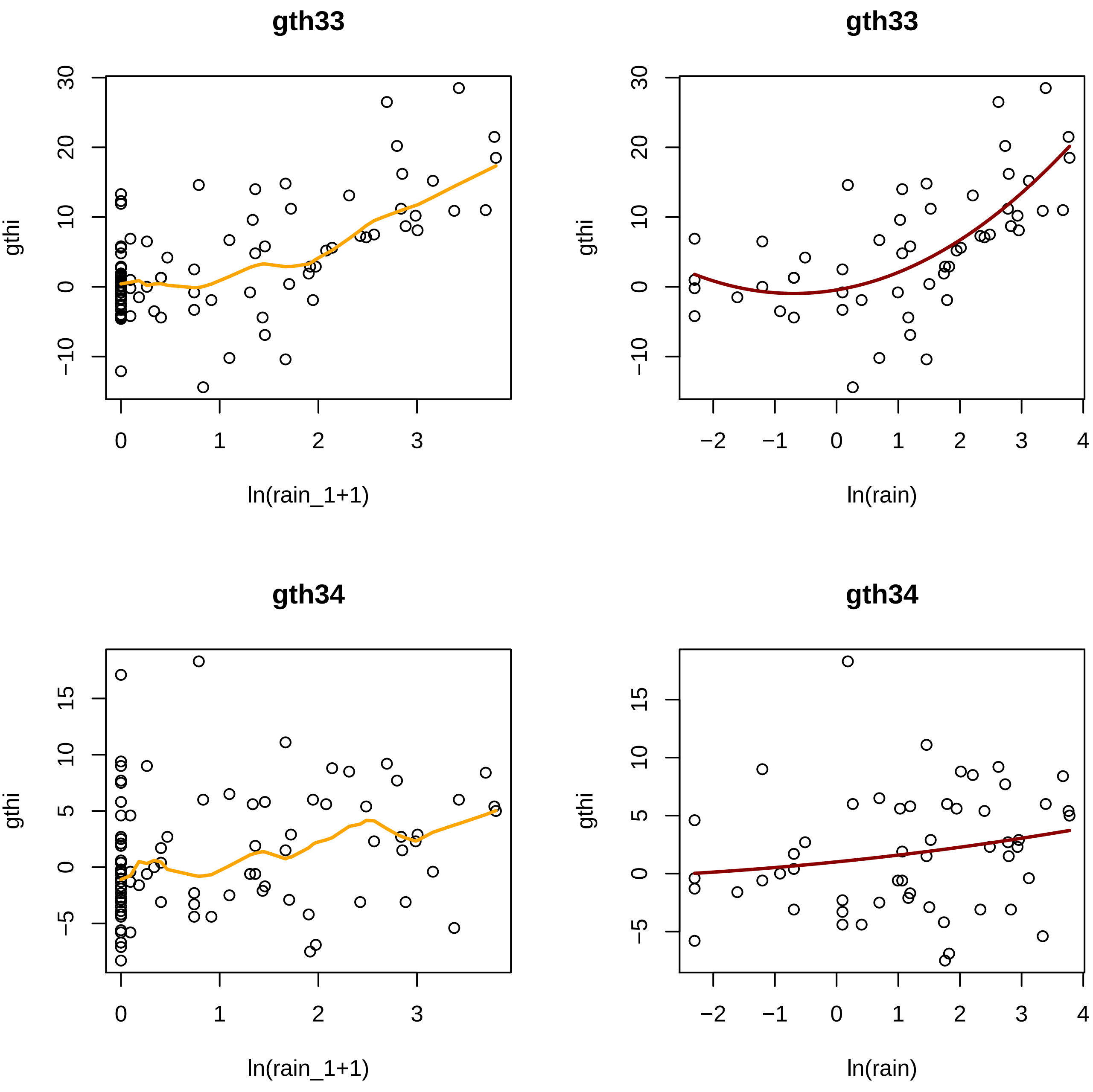

gth33
gth33
gth34
gth34
gthi
ln(rain_1+1)
ln(rain)

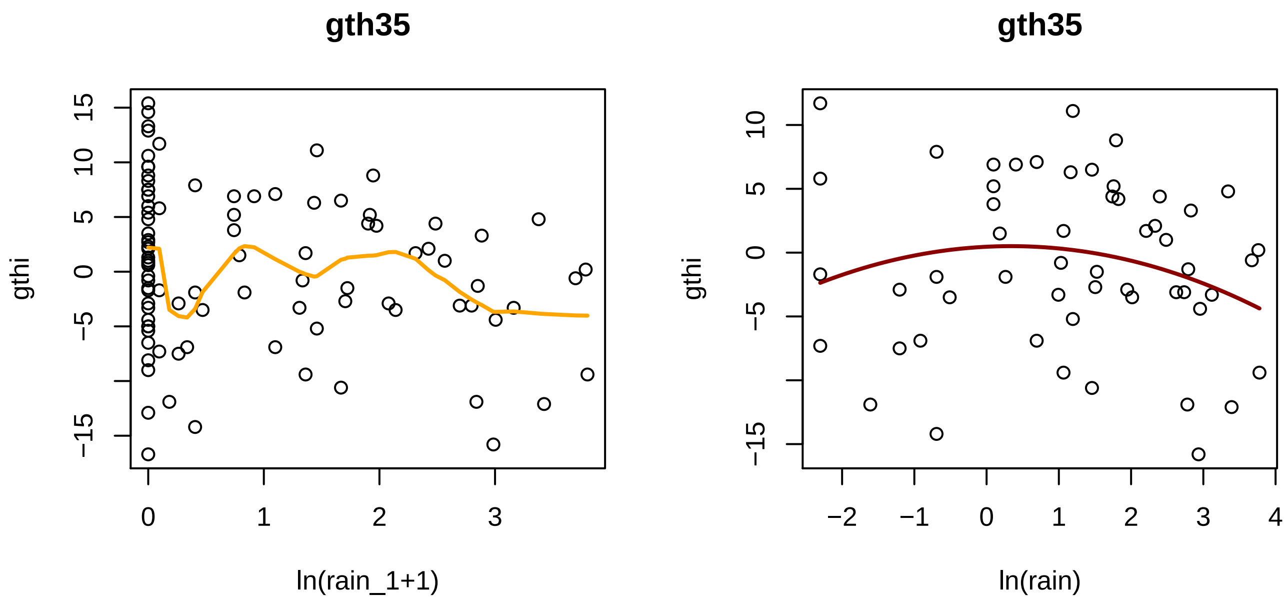
gth35
gthi
ln(rain_1+1)
15
10
5
0
−5
−15
0
1
2
3
gth35
gthi
ln(rain)
10
5
0
−5
−15
−2
−1
0
1
2
3
4

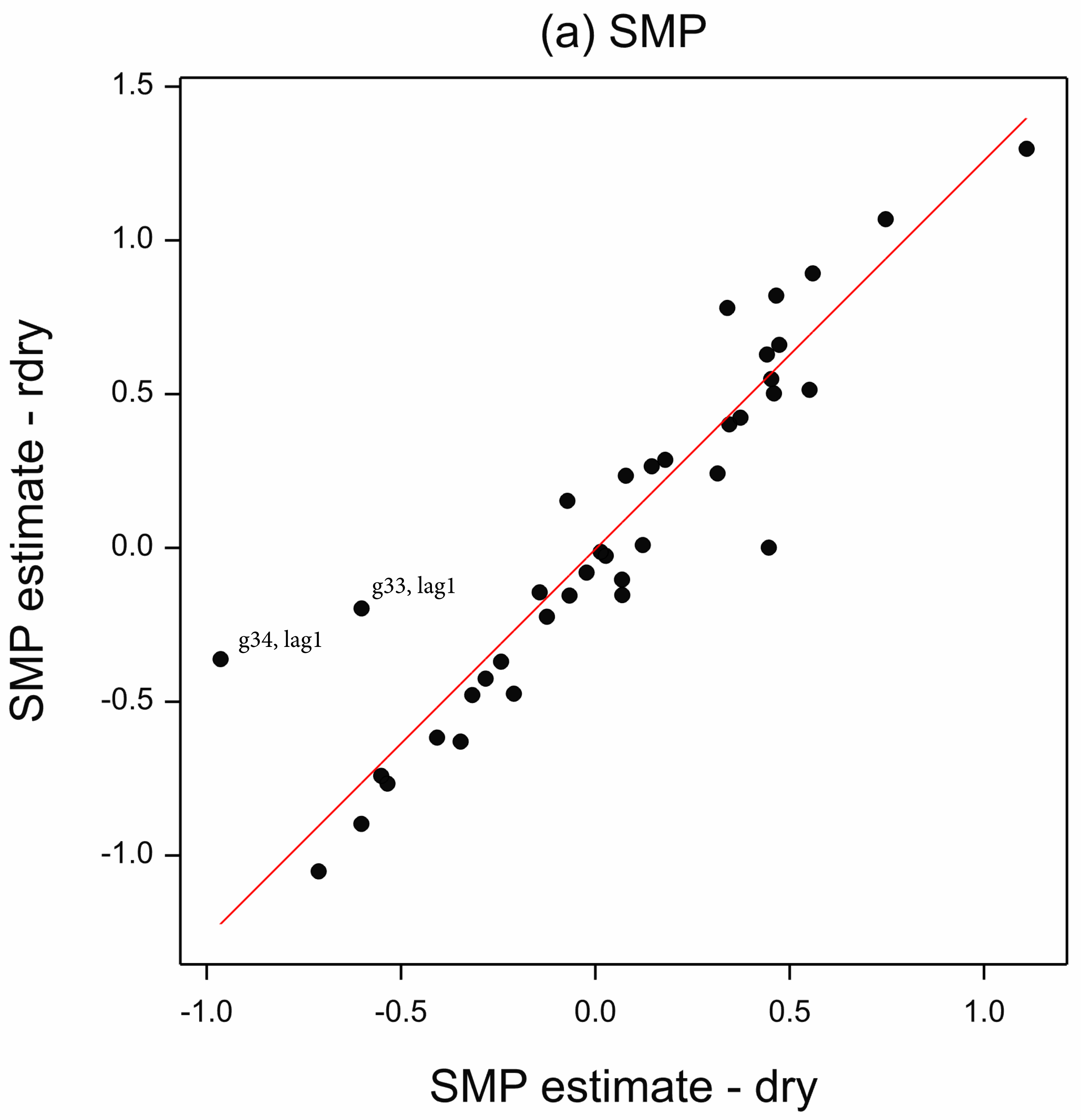

S4 Appendix: Fig C. Estimates of (a) SMP, and (b) TEMP, coefficients from the GLS-arima single-term model fits for the 13 dendrobands recorded in the dry period; each for three lags (0, 1 and 2 days), plotted for analyses that used the original daily growth increments, gthi, ('dry') versus those that used the residuals from fitting gthi to rainfall ('rdry'). The two most important outliers in each case are identified.

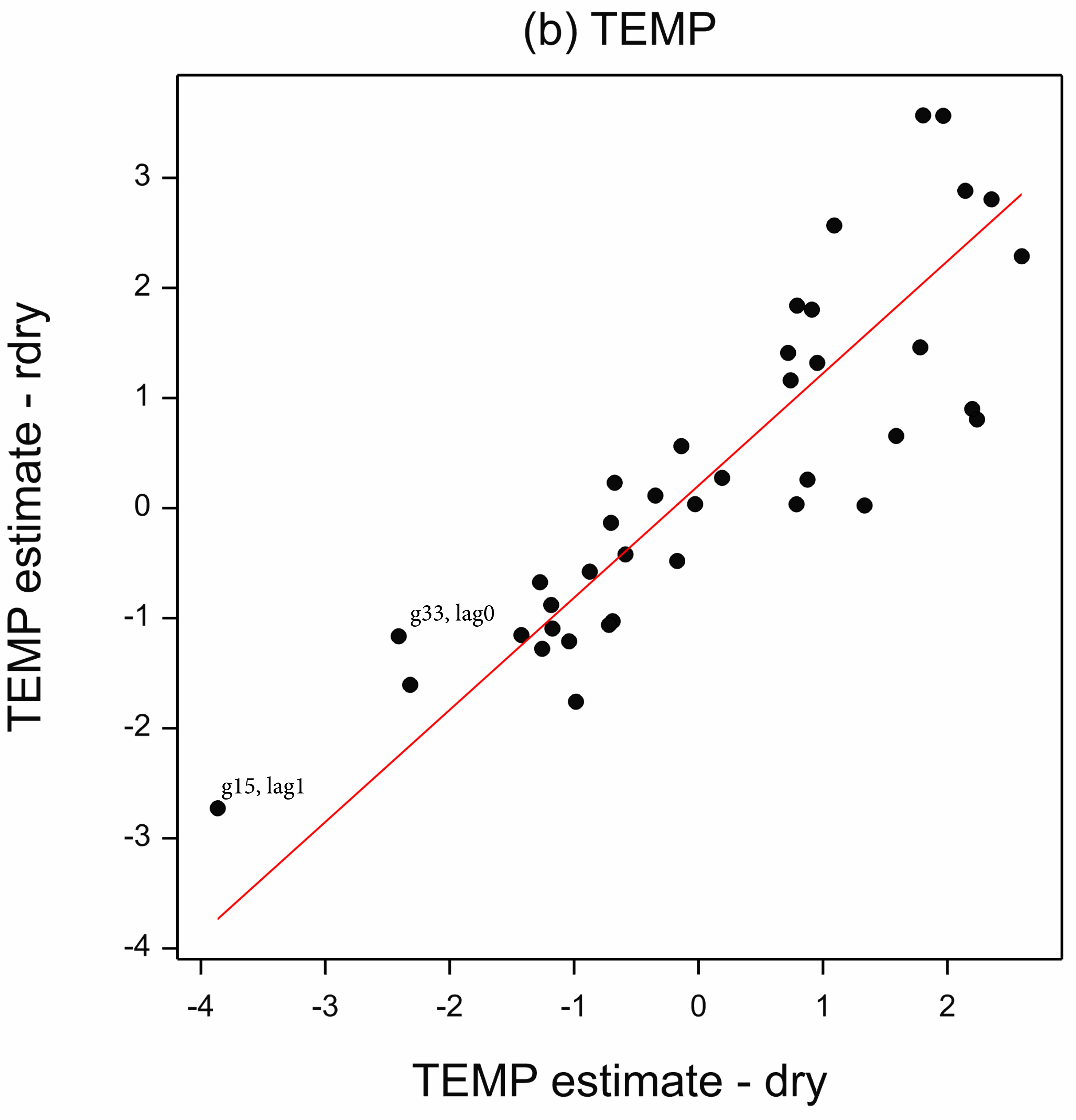

(b) TEMP
TEMP estimate - rdry
TEMP estimate - dry
g33, lag0
g15, lag1
3
2
1
0
-1
-2
-3
-4
-4
-3
-2
-1
0
1
2
