# Supplementary material for: Stem girth changes in response to soil water potential in lowland dipterocarp forest in Borneo: An individualistic time-series analysis
Source: PLoS One. 2022 Jun 30;17(6):e0270140. doi: 10.1371/journal.pone.0270140 (PMC9246238; doi:10.1371/journal.pone.0270140)
Supplement: S5 Appendix — (PDF) [file pone.0270140.s005.pdf]

S5 Appendix: Table A. Tables of *t*-values and their probability levels for three time lags (0, 1, and 2 days) for GLS-regression coefficients (slopes) of daily stem girth increment versus soil moisture potential (SMP). In these regression fits SMP was the only independent variable. The coefficients and their standard errors are represented in Fig 4 of the main paper.

season	gth	spec	t(est)_0	P(t)_0	t(est)_1	P(t)_1	t(est)_2	P(t)_2
w	g11	Mw	3.848	0.00013	6.963	<0.00001	5.359	<0.00001
w	g12	Sf	0.673	0.50102	−5.080	<0.00001	−2.071	0.03879
w	g14	Lb	0.150	0.44100	−0.277	0.39100	0.216	0.41400
w	g15	Sf	2.464	0.01406	6.652	<0.00001	5.371	<0.00001
w	g22	Pm	−7.239	<0.00001	−6.921	<0.00001	−5.697	<0.00001
w	g23	Dm	0.311	0.37800	3.071	0.00100	2.434	0.00800
w	g24	Mw	−2.710	0.00693	−2.128	0.03375	−1.289	0.19803
w	g25	Sf	1.735	0.08324	6.190	<0.00001	4.667	<0.00001
w	g31	Mw	−4.140	0.00004	−4.180	0.00003	−3.752	0.00019
w	g32	Sp	−0.289	0.77268	−0.293	0.76926	−0.170	0.86508
w	g33	Lb	−4.972	<0.00001	−4.365	0.00002	−2.450	0.01460
w	g34	Dm	−3.237	0.00128	−4.288	0.00002	−3.848	0.00013
w	g35	Dm	−0.216	0.82876	4.288	0.00002	4.464	0.00001
w	g41	Sf	−2.404	0.00900	−2.736	0.00300	−2.662	0.00400
w	g42	Dm	4.069	0.00005	6.917	<0.00001	6.589	<0.00001
w	g43	Pm	−5.920	<0.00001	−5.884	<0.00001	−5.673	<0.00001
w	g44	Sp	−6.367	<0.00001	−8.258	<0.00001	−5.722	<0.00001
w	g45	Pm	−4.508	0.00001	−5.458	<0.00001	−4.787	<0.00001
d	g11	Mw	−1.176	0.24254	−0.825	0.41135	0.079	0.93696
d	g12	Sf	0.291	0.77178	−0.268	0.78921	−0.872	0.38520
d	g14	Lb	−2.851	0.00533	−2.516	0.01355	−2.718	0.00780
d	g15	Sf	2.335	0.02162	5.258	<0.00001	5.035	<0.00001
d	g22	Pm	−2.599	0.01090	−1.101	0.27367	−0.093	0.92650
d	g23	Dm	3.155	0.00217	3.320	0.00130	1.492	0.13921
d	g24	Mw	−3.838	0.00023	−3.816	0.00025	−3.477	0.00078
d	g25	Sf	4.995	<0.00001	7.109	<0.00001	6.225	<0.00001
d	g31	Mw	−4.416	0.00003	−4.117	0.00008	−3.595	0.00053
d	g32	Sp	4.034	0.00011	6.049	<0.00001	4.945	<0.00001
d	g33	Lb	0.006	0.99555	−1.128	0.26210	0.998	0.32082
d	g34	Dm	−1.254	0.21308	−3.205	0.00186	−2.111	0.03750
d	g35	Dm	2.384	0.01920	3.006	0.00342	2.921	0.00440

Coefficients significant at the familywise α ≤ 0.05, 0.01 and 0.002 levels are background colored in increasing intensities of green for the wet series and orange for the dry one. Simple Bonferroni adjustment was applied by dividing the family α-value by the number of bands/trees per series, α/18 and α/13 respectively. The adjusted α's for wet were *P* = 0.00278, 0.00056 and 0.00011; and for dry *P* = 0.00385, 0.00077 and 0.00015.

S5 Appendix: Table B. Tables of *t*-values and their probability levels for three time lags (0, 1, and 2 days) for GLS-regressions coefficients (slopes) of daily girth increment versus ambient temperature (TEMP). In these regression fits TEMP was the only independent variable. The coefficients and their standard errors are represented in Fig 5 of the main paper.

season	gth	spec	t(est)_0	P(t)_0	t(est)_1	P(t)_1	t(est)_2	P(t)_2
w	g11	Mw	3.053	0.00238	−5.143	<0.00001	−1.658	0.09783
w	g12	Sf	−5.737	<0.00001	1.786	0.07462	−1.383	0.16725
w	g14	Lb	−0.980	0.32860	0.490	0.62440	−0.461	0.64500
w	g15	Sf	4.773	<0.00001	−4.112	0.00005	−0.514	0.60742
w	g22	Pm	6.084	<0.00001	2.140	0.03293	2.585	0.01004
w	g23	Dm	5.228	0.00001	−2.816	0.00541	−0.482	0.63040
w	g24	Mw	4.423	0.00001	1.076	0.28239	1.219	0.22347
w	g25	Sf	6.309	<0.00001	−5.214	<0.00001	0.444	0.65739
w	g31	Mw	4.869	<0.00001	1.077	0.28185	3.234	0.00129
w	g32	Sp	1.485	0.13811	−0.416	0.67789	−1.753	0.08008
w	g33	Lb	6.324	<0.00001	0.934	0.35072	1.591	0.11212
w	g34	Dm	0.891	0.37355	1.013	0.31146	1.985	0.04761
w	g35	Dm	6.091	<0.00001	−0.750	0.45387	−0.114	0.90965
w	g41	Sf	3.037	0.00275	0.057	0.95460	1.357	0.17660
w	g42	Dm	7.662	<0.00001	−4.963	<0.00001	−3.780	0.00017
w	g43	Pm	3.047	0.00242	−0.172	0.86375	1.337	0.18176
w	g44	Sp	1.880	0.06064	1.906	0.05714	2.691	0.00733
w	g45	Pm	1.564	0.11838	2.439	0.01504	2.088	0.03722
d	g11	Mw	0.523	0.60215	0.080	0.93658	1.259	0.21116
d	g12	Sf	−2.754	0.00704	−1.555	0.12327	−1.775	0.07903
d	g14	Lb	4.276	0.00004	1.367	0.17472	3.497	0.00071
d	g15	Sf	1.275	0.20538	−4.207	0.00006	−2.510	0.01375
d	g22	Pm	6.616	<0.00001	2.758	0.00703	3.064	0.00288
d	g23	Dm	−2.636	0.00988	−3.077	0.00276	−3.414	0.00096
d	g24	Mw	4.483	0.00002	0.053	0.95807	4.551	0.00002
d	g25	Sf	2.321	0.02253	−2.494	0.01442	−0.870	0.38669
d	g31	Mw	4.054	0.00011	2.386	0.01911	3.921	0.00017
d	g32	Sp	0.887	0.37748	−2.969	0.00382	−3.847	0.00022
d	g33	Lb	−3.094	0.00263	3.015	0.00333	0.088	0.92986
d	g34	Dm	−0.543	0.58859	1.056	0.29367	0.456	0.64942
d	g35	Dm	1.683	0.09573	−3.223	0.00176	−2.822	0.00586

See footnote to Table S1 for colour coding.

S5 Appendix: Table C. Tables of *t*-values and their probability levels for two time lags (0 and 1 days) for GLS-regression SMP coefficients (slopes) of daily stem girth increment versus soil moisture potential (SMP) and ambient temperature (TEMP). In these regression fits there were two independent terms, SMP and TEMP, but no interaction term. The coefficients and their standard errors are represented in Fig A of S6 Appendix.

season	gth	spec	t(est)_00	P(t)_00	t(est)_01	P(t)_01	t(est)_10	P(t)_10	t(est)_11	P(t)_11
w	g11	Mw	5.294	<0.00001	2.266	0.02385	7.854	<0.00001	5.455	<0.00001
w	g12	Sf	−1.285	0.19918	1.177	0.23985	−4.237	0.00003	−2.768	0.00583
w	g14	Lb	−0.048	0.96200	0.191	0.84900	−0.114	0.90940	−0.211	0.83340
w	g15	Sf	4.582	0.00001	1.306	0.19205	8.188	<0.00001	5.592	<0.00001
w	g22	Pm	−5.996	<0.00001	−6.894	<0.00001	−6.124	<0.00001	−6.529	<0.00001
w	g23	Dm	1.854	0.06534	−0.462	0.64440	3.665	0.00033	2.279	0.02386
w	g24	Mw	−1.124	0.26152	−2.502	0.01264	−0.957	0.33879	−1.865	0.06270
w	g25	Sf	4.331	0.00002	0.195	0.84545	7.759	<0.00001	4.727	<0.00001
w	g31	Mw	−2.463	0.01410	−4.177	0.00003	−2.648	0.00833	−4.124	0.00004
w	g32	Sp	0.151	0.88012	−0.393	0.69457	−0.231	0.81748	−0.444	0.65720
w	g33	Lb	−3.518	0.00047	−4.773	<0.00001	−4.083	0.00005	−4.128	0.00004
w	g34	Dm	−3.172	0.00160	−3.090	0.00210	−4.340	0.00002	−4.175	0.00003
w	g35	Dm	2.199	0.02828	−0.547	0.58483	5.873	<0.00001	4.367	0.00002
w	g41	Sf	−1.603	0.11062	−2.564	0.01117	−1.892	0.06004	−2.768	0.06228
w	g42	Dm	6.455	<0.00001	1.535	0.12525	9.109	<0.00001	5.386	<0.00001
w	g43	Pm	−5.237	<0.00001	−6.333	<0.00001	−5.396	<0.00001	−6.253	<0.00001
w	g44	Sp	−4.834	<0.00001	−6.167	<0.00001	−8.113	<0.00001	−7.876	<0.00001
w	g45	Pm	−4.288	0.00002	−3.978	0.00008	−5.435	<0.00001	−4.975	<0.00001
d	g11	Mw	−1.075	0.28530	−1.294	0.19871	−0.688	0.49343	−0.870	0.38666
d	g12	Sf	−0.874	0.38439	0.767	0.44491	−1.271	0.20695	−0.494	0.62216
d	g14	Lb	−1.809	0.07354	−2.581	0.01139	−1.893	0.06142	−2.208	0.02966
d	g15	Sf	2.234	0.02782	0.888	0.37695	5.460	<0.00001	4.152	0.00007
d	g22	Pm	−0.539	0.59131	−1.779	0.07864	0.727	0.46910	−0.156	0.87676
d	g23	Dm	2.803	0.00620	2.187	0.03137	3.587	0.00054	2.479	0.01502
d	g24	Mw	−2.823	0.00585	−4.448	0.00002	−2.347	0.02114	−3.096	0.00261

d	g25	Sf	6.488	<0.00001	3.111	0.00250	8.229	<0.00001	5.208	<0.00001
d	g31	Mw	−2.968	0.00384	−3.818	0.00025	−2.678	0.00881	−3.349	0.00119
d	g32	Sp	3.993	0.00013	2.970	0.00382	5.977	<0.00001	5.132	<0.00001
d	g33	Lb	−1.375	0.17262	0.549	0.58457	−1.147	0.25429	−0.505	0.61508
d	g34	Dm	−1.298	0.19750	−1.404	0.16382	−3.194	0.00194	−2.995	0.00354
d	g35	Dm	3.091	0.00266	0.567	0.57205	3.565	0.00059	1.782	0.07819

See footnote to Table S1 for colour coding.

S5 Appendix: Table D. Tables of *t*-values and their probability levels for two time lags (0 and 1 days) for GLS-regression TEMP coefficients (slopes) of daily stem girth increment versus soil moisture potential (SMP) and ambient temperature (TEMP). In these regression fits there were two independent terms, SMP and TEMP, but no interaction term. The coefficients and their standard errors are represented in Fig B of S6 Appendix.

season	gth	spec	t(est)_00	P(t)_00	t(est)_01	P(t)_01	t(est)_10	P(t)_10	t(est)_11	P(t)_11
w	g11	Mw	4.724	<0.00001	−4.134	0.00004	4.506	0.00001	−2.930	0.00353
w	g12	Sf	−5.816	<0.00001	2.047	0.04115	−6.492	<0.00001	0.825	0.40975
w	g14	Lb	−1.022	0.30820	0.406	0.68560	−1.078	0.28220	0.356	0.72200
w	g15	Sf	6.226	<0.00001	−3.521	0.00047	6.734	<0.00001	−1.988	0.04732
w	g22	Pm	3.898	0.00011	0.188	0.85083	4.973	<0.00001	0.594	0.55255
w	g23	Dm	5.649	0.00001	−2.792	0.00581	5.600	0.00001	−1.928	0.05544
w	g24	Mw	3.609	0.00034	−0.089	0.92889	4.004	0.00007	0.216	0.82887
w	g25	Sf	7.500	<0.00001	−4.947	<0.00001	7.798	<0.00001	−3.269	0.00115
w	g31	Mw	3.249	0.00123	−1.199	0.23110	3.465	0.00057	−0.966	0.33458
w	g32	Sp	1.463	0.14414	−0.492	0.62285	1.483	0.13871	−0.532	0.59465
w	g33	Lb	5.128	<0.00001	−0.235	0.81416	6.101	<0.00001	0.104	0.91757
w	g34	Dm	−0.493	0.62233	−0.267	0.78925	−0.555	0.57918	−0.609	0.54247
w	g35	Dm	6.486	<0.00001	−0.916	0.35999	7.309	<0.00001	1.023	0.30668
w	g41	Sf	2.012	0.04570	−1.094	0.27540	2.339	0.02042	−1.006	0.31600
w	g42	Dm	8.920	<0.00001	−3.594	0.00036	9.059	<0.00001	−2.295	0.02209
w	g43	Pm	1.134	0.25745	−2.366	0.01834	1.192	0.23372	−2.321	0.02068
w	g44	Sp	0.202	0.84038	0.399	0.69014	1.164	0.24511	0.155	0.87703
w	g45	Pm	−0.450	0.65270	0.544	0.58645	−0.417	0.67648	0.151	0.88026
d	g11	Mw	−0.053	0.95764	−0.551	0.58265	0.229	0.81970	−0.298	0.76669
d	g12	Sf	−2.869	0.00507	−1.134	0.25976	−3.034	0.00321	−1.592	0.11464
d	g14	Lb	3.124	0.00236	0.675	0.50103	3.648	0.00043	0.411	0.68173
d	g15	Sf	1.739	0.08536	−3.701	0.00036	1.860	0.06603	−2.858	0.00525
d	g22	Pm	6.041	<0.00001	2.078	0.04053	6.586	<0.00001	2.324	0.02239
d	g23	Dm	−0.465	0.64331	−2.027	0.04557	−1.762	0.08144	−2.075	0.04080
d	g24	Mw	2.801	0.00624	−2.613	0.01051	3.715	0.00035	1.170	0.24501

d	g25	Sf	1.894	0.06150	−1.939	0.05562	1.824	0.07139	−1.733	0.08654
d	g31	Mw	2.834	0.00568	0.427	0.67064	3.196	0.00193	1.003	0.31858
d	g32	Sp	0.959	0.34034	−2.274	0.02533	0.701	0.48514	−1.757	0.08231
d	g33	Lb	−3.344	0.00120	2.927	0.00434	−3.167	0.00210	2.769	0.00683
d	g34	Dm	−0.769	0.44409	0.938	0.35094	−0.618	0.53787	0.111	0.91214
d	g35	Dm	2.903	0.00464	−2.516	0.01365	2.611	0.01058	−2.163	0.03320

See footnote to Table S1 for colour coding.

S5 Appendix: Table E1. Tables of *t*–values and their probability levels for two time lags (0 and 1 days) for GLS–regression SMP*TEMP interaction of daily stem girth increment, and their coefficients and standard errors, versus soil moisture potential (SMP) and ambient temperature (TEMP). In these regression fits there were two independent terms, SMP and TEMP, plus the interaction term. Here SMP had no lag and TEMP had either no lag (0, 0) or was lagged by 1 day also (0, 1). Coefficients of SMP and TEMP for this particular regression model are not reported in the paper.

season	gth	spec	est_00	se_00	t(est)_00	P(t)_00	est_01	se_01	t(est)_01	P(t)_01
w	g11	Mw	−0.1426	0.0550	−2.5912	0.00982	0.0352	0.0583	0.6040	0.54611
w	g12	Sf	0.0475	0.0819	0.5807	0.56166	−0.0060	0.0861	−0.0696	0.94455
w	g14	Lb	0.0679	0.8327	0.5896	0.55600	0.2917	0.9048	1.1254	0.26200
w	g15	Sf	−0.0559	0.0589	−0.9505	0.34226	0.0245	0.0603	0.4064	0.68459
w	g22	Pm	−0.4455	0.1877	−2.3734	0.01805	−0.2212	0.2029	−1.0898	0.27640
w	g23	Dm	−0.0215	0.4146	−0.1000	0.92040	−0.2266	0.4463	−0.5366	0.59220
w	g24	Mw	−0.0283	0.0311	−0.9094	0.36354	0.0215	0.0329	0.6512	0.51522
w	g25	Sf	−0.1791	0.0667	−2.6839	0.00750	0.0231	0.0722	0.3201	0.74903
w	g31	Mw	−0.0533	0.0228	−2.3435	0.01946	0.0528	0.0257	2.0559	0.04026
w	g32	Sp	−0.0327	0.0311	−1.0499	0.29425	0.0227	0.0341	0.6644	0.50674
w	g33	Lb	−0.0405	0.0200	−2.0230	0.04356	0.0167	0.0226	0.7375	0.46112
w	g34	Dm	−0.0387	0.0274	−1.4091	0.15938	0.0296	0.0303	0.9763	0.32936
w	g35	Dm	−0.0608	0.0519	−1.1719	0.24176	0.0354	0.0573	0.6186	0.53644
w	g41	Sf	−0.1200	0.1053	−0.0819	0.93480	−0.0501	0.1050	0.1927	0.84740
w	g42	Dm	−0.0167	0.0197	−0.8472	0.39725	0.0009	0.0202	0.0463	0.96310
w	g43	Pm	−0.0420	0.0825	−0.5090	0.61099	0.0588	0.0897	0.6550	0.51273
w	g44	Sp	−0.0217	0.0347	−0.6263	0.53135	0.0662	0.0538	1.2306	0.21899
w	g45	Pm	−0.0221	0.0361	−0.6116	0.54107	0.0016	0.0301	0.0540	0.95697
d	g11	Mw	0.0175	0.0141	1.237	0.21931	0.0171	0.0141	1.215	0.22732
d	g12	Sf	−0.0107	0.0076	−1.405	0.16320	−0.0093	0.0081	−1.158	0.24972
d	g14	Lb	−0.0112	0.0173	−0.648	0.51862	−0.0183	0.0150	−1.219	0.22608
d	g15	Sf	0.1079	0.0174	6.198	<0.00001	0.1007	0.0167	6.024	<0.00001
d	g22	Pm	0.0293	0.0126	2.319	0.02269	0.0542	0.0127	4.263	0.00005
d	g23	Dm	0.0371	0.0088	4.210	0.00006	0.0158	0.0108	1.458	0.14828

d	g24	Mw	0.0078	0.0106	0.740	0.46143	0.0056	0.0101	0.549	0.58450
d	g25	Sf	0.0585	0.0173	3.388	0.00105	0.0569	0.0171	3.335	0.00125
d	g31	Mw	0.0162	0.0251	0.645	0.52034	0.0019	0.0268	0.070	0.94426
d	g32	Sp	0.0390	0.0075	5.175	<0.00001	0.0381	0.0073	5.204	<0.00001
d	g33	Lb	−0.0044	0.0114	−0.384	0.70216	−0.0058	0.0116	−0.502	0.61720
d	g34	Dm	−0.0254	0.0068	−3.757	0.00031	−0.0249	0.0068	−3.677	0.00040
d	g35	Dm	0.0199	0.0105	1.905	0.06002	0.0232	0.0106	2.180	0.03188

See footnote to Table S1 for colour coding.

S5 Appendix: Table E2. Tables of t-values and their probability levels for two time lags (0 and 1 days) for GLS–regression SMP*TEMP interaction coefficients of daily stem girth increment, and their coefficients and standard errors, versus soil moisture potential (SMP) and ambient temperature (TEMP). In these regression fits there were two independent terms, SMP and TEMP, plus the interaction term. Here SMP had a lag of 1 day and TEMP had either no lag (1, 0) or was lagged by 1 day also (1, 1). Coefficients of SMP and TEMP for this particular regression model are not reported in the paper.

season	gth	spec	est_10	se_10	t(est)_10	P(t)_10	est_11	se_11	t(est)_11	P(t)_11
w	g11	Mw	−0.1001	0.0455	−2.2023	0.02806	0.1089	0.0426	2.5544	0.01091
w	g12	Sf	0.0356	0.0510	0.6983	0.48528	−0.0373	0.0615	−0.6069	0.54417
w	g14	Lb	−0.2908	0.8124	−0.3634	0.71680	0.2488	0.8466	1.0011	0.31820
w	g15	Sf	−0.0043	0.0198	−0.2150	0.82987	0.0358	0.0318	1.1239	0.26155
w	g22	Pm	−0.3796	0.1787	−2.1244	0.03419	−0.3102	0.1864	−1.6639	0.09683
w	g23	Dm	−0.1353	0.4142	−0.7092	0.47920	−0.1500	0.4217	−0.2582	0.79660
w	g24	Mw	−0.0294	0.0310	−0.9479	0.34360	0.0106	0.0300	0.3517	0.72520
w	g25	Sf	−0.2031	0.0728	−2.7914	0.00543	0.1647	0.0672	2.4490	0.01464
w	g31	Mw	−0.0453	0.0245	−1.8510	0.06472	0.0341	0.0197	1.7290	0.08437
w	g32	Sp	−0.0202	0.0275	−0.7352	0.46255	0.0457	0.0117	3.9192	0.00010
w	g33	Lb	−0.0154	0.0054	−2.8290	0.00484	0.0168	0.0065	2.5644	0.01060
w	g34	Dm	−0.0288	0.0216	−1.3324	0.18329	0.0349	0.0229	1.5218	0.12863
w	g35	Dm	−0.0784	0.0720	−1.0893	0.27652	0.0674	0.0709	0.9501	0.34248
w	g41	Sf	−0.0939	0.0972	−0.1452	0.88480	−0.0404	0.0930	0.2820	0.77820
w	g42	Dm	−0.0262	0.0186	−1.4051	0.16055	0.0152	0.0191	0.7976	0.42547
w	g43	Pm	0.0015	0.0252	0.0586	0.95331	−0.0179	0.0236	−0.7578	0.44892
w	g44	Sp	0.0005	0.0085	0.0564	0.95503	−0.0040	0.0097	−0.4079	0.68347
w	g45	Pm	−0.0023	0.0098	−0.2307	0.81763	−0.0029	0.0112	−0.2602	0.79483
d	g11	Mw	0.0292	0.0856	0.341	0.73414	−0.0044	0.0345	−0.127	0.89938
d	g12	Sf	−0.0202	0.0463	−0.435	0.66448	0.0355	0.0192	1.848	0.06779
d	g14	Lb	−0.0587	0.1160	−0.506	0.61422	−0.1545	0.0428	−3.610	0.00049
d	g15	Sf	−0.4838	0.1077	−4.490	0.00002	−0.1305	0.0427	−3.053	0.00295
d	g22	Pm	−0.0227	0.0799	−0.285	0.77645	−0.2042	0.0330	−6.197	<0.00001
d	g23	Dm	−0.1528	0.0665	−2.299	0.02383	0.0187	0.0262	0.714	0.47679

d	g24	Mw	−0.1614	0.0744	−2.169	0.03271	−0.1123	0.0298	−3.774	0.00029
d	g25	Sf	−0.2257	0.1096	−2.059	0.04243	−0.0960	0.0420	−2.287	0.02457
d	g31	Mw	−0.1181	0.1628	−0.725	0.47019	−0.2132	0.0631	−3.378	0.00108
d	g32	Sp	−0.1820	0.0489	−3.720	0.00035	−0.0422	0.0182	−2.316	0.02287
d	g33	Lb	−0.1329	0.0695	−1.913	0.05897	0.0616	0.0260	2.364	0.02026
d	g34	Dm	0.0586	0.0453	1.293	0.19947	0.0203	0.0164	1.238	0.21910
d	g35	Dm	0.0318	0.0705	0.451	0.65329	−0.0881	0.0267	−3.299	0.00140

See footnote to Table S1 for color coding.
