# Supplementary material for: Stem girth changes in response to soil water potential in lowland dipterocarp forest in Borneo: An individualistic time-series analysis
Source: PLoS One. 2022 Jun 30;17(6):e0270140. doi: 10.1371/journal.pone.0270140 (PMC9246238; doi:10.1371/journal.pone.0270140)

S6 Appendix: Fig A. Mean ± SE coefficients (slopes) for SMP of *gthi* regressed on soil moisture potential, SMP, at 0- and 1-day lags, with logger temperature, TEMP, at 0- and 1-day lags, in the wet (green bars) and dry (orange bars) periods for the 18 trees taken for the time-series analyses. Predictions of dry-period estimates for station 4 (see text for explanation, and Table E in S1 Appendix) are shown in blue. Bands are listed in Table 4 (main text) with their individual tree codes.

S6 Appendix: Fig B. Mean ± SE coefficients (slopes) for TEMP of *gthi* regressed on soil moisture potential, SMP, at 0- and 1-day lags, with logger temperature, TEMP, at 0- and 1-day lags, in the wet (green bars) and dry (orange bars) periods for the 18 trees taken for the time-series analyses. Predictions of dry-period estimates for station 4 (see text for explanation, and Table E in S1 Appendix) are shown in blue. Bands are listed in Table 4 (main text) with their individual tree codes.

Fig. A

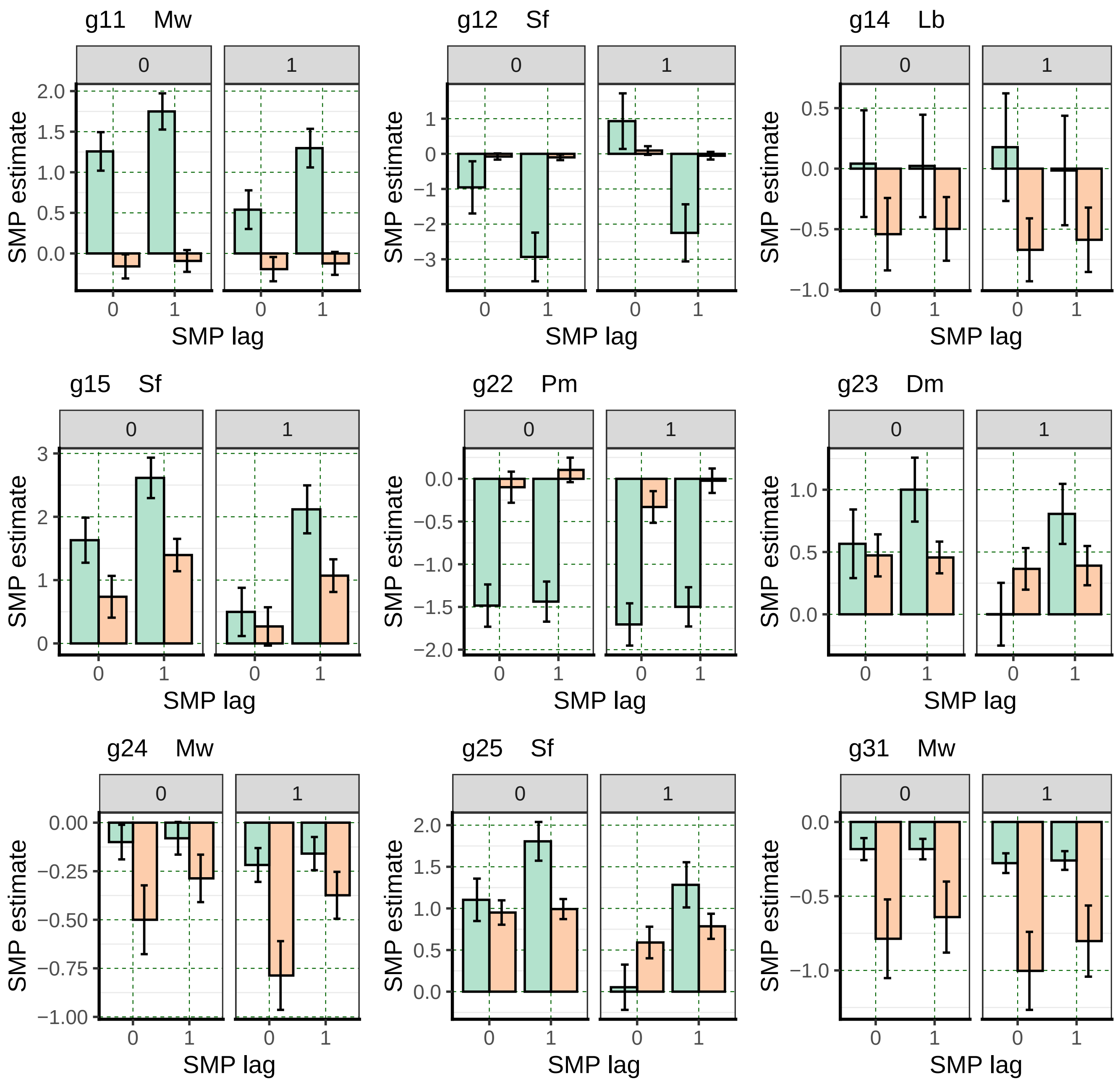

g11 Mw
g12 Sf
g14 Lb
g15 Sf
g22 Pm
g23 Dm
g24 Mw
g25 Sf
g31 Mw
SMP estimate
SMP lag

Fig A, continued

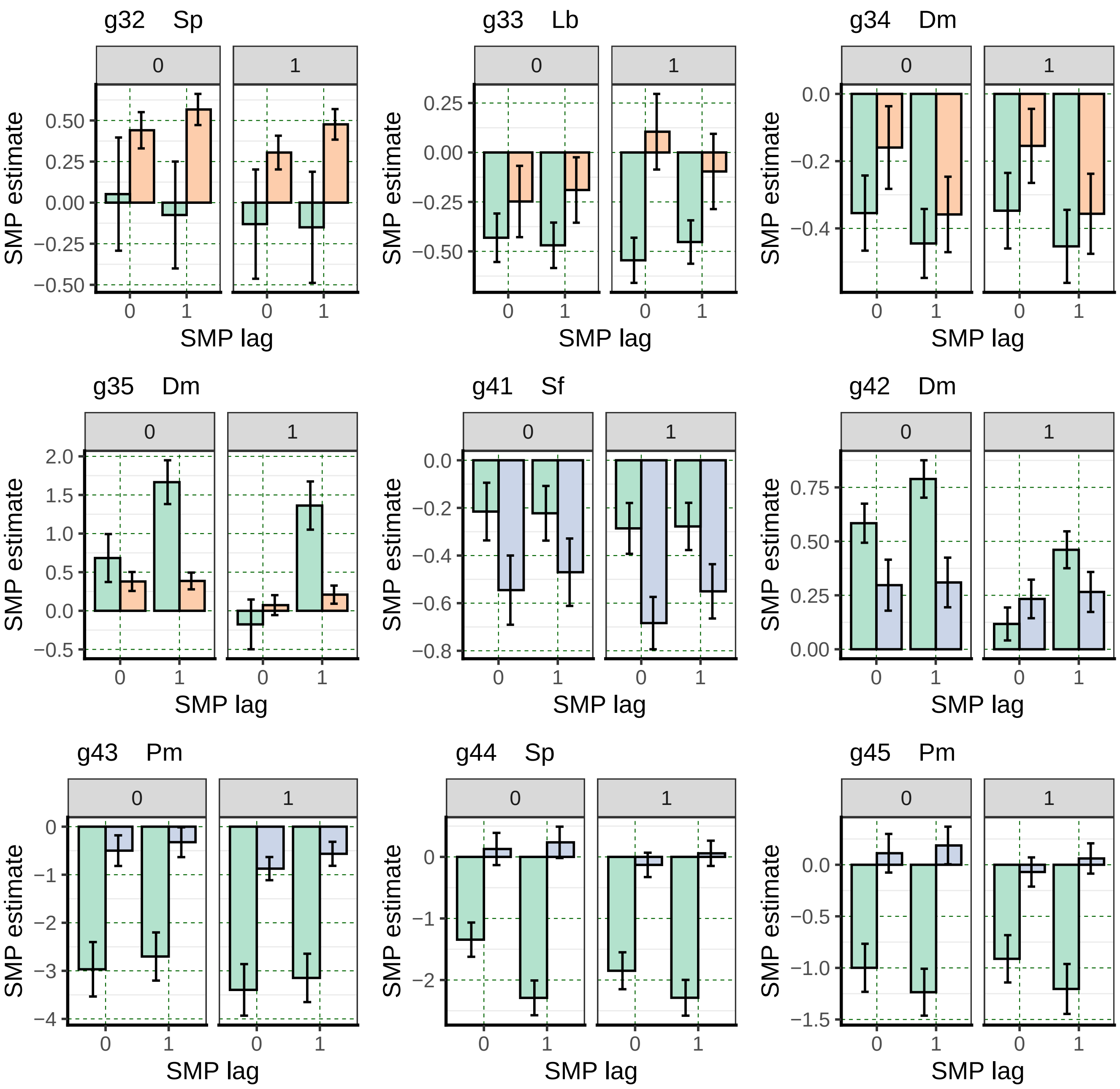

Fig B

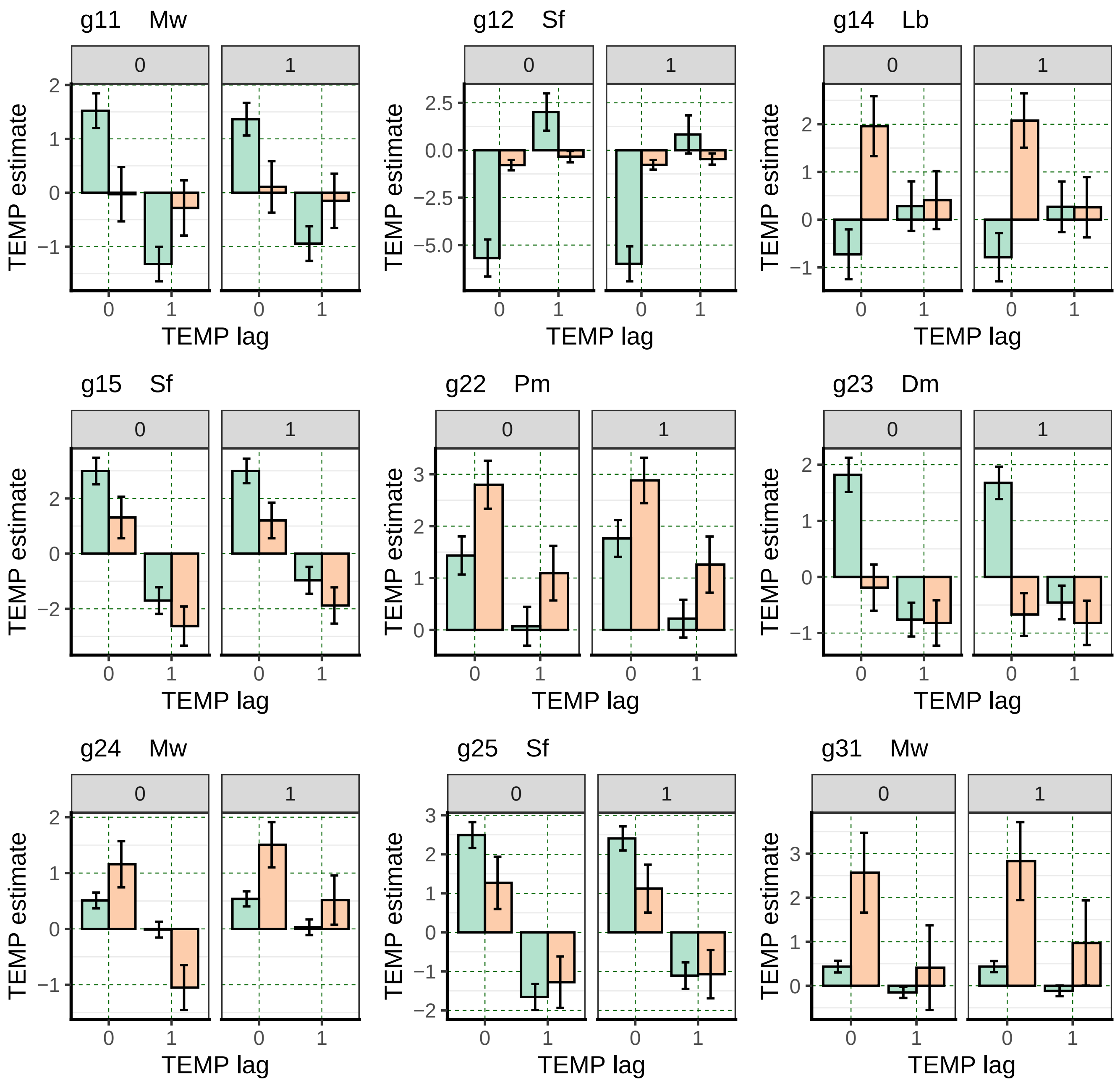
g11 Mw
g12 Sf
g14 Lb
g15 Sf
g22 Pm
g23 Dm
g24 Mw
g25 Sf
g31 Mw
0
1
TEMP estimate
TEMP lag

Fig B, continued

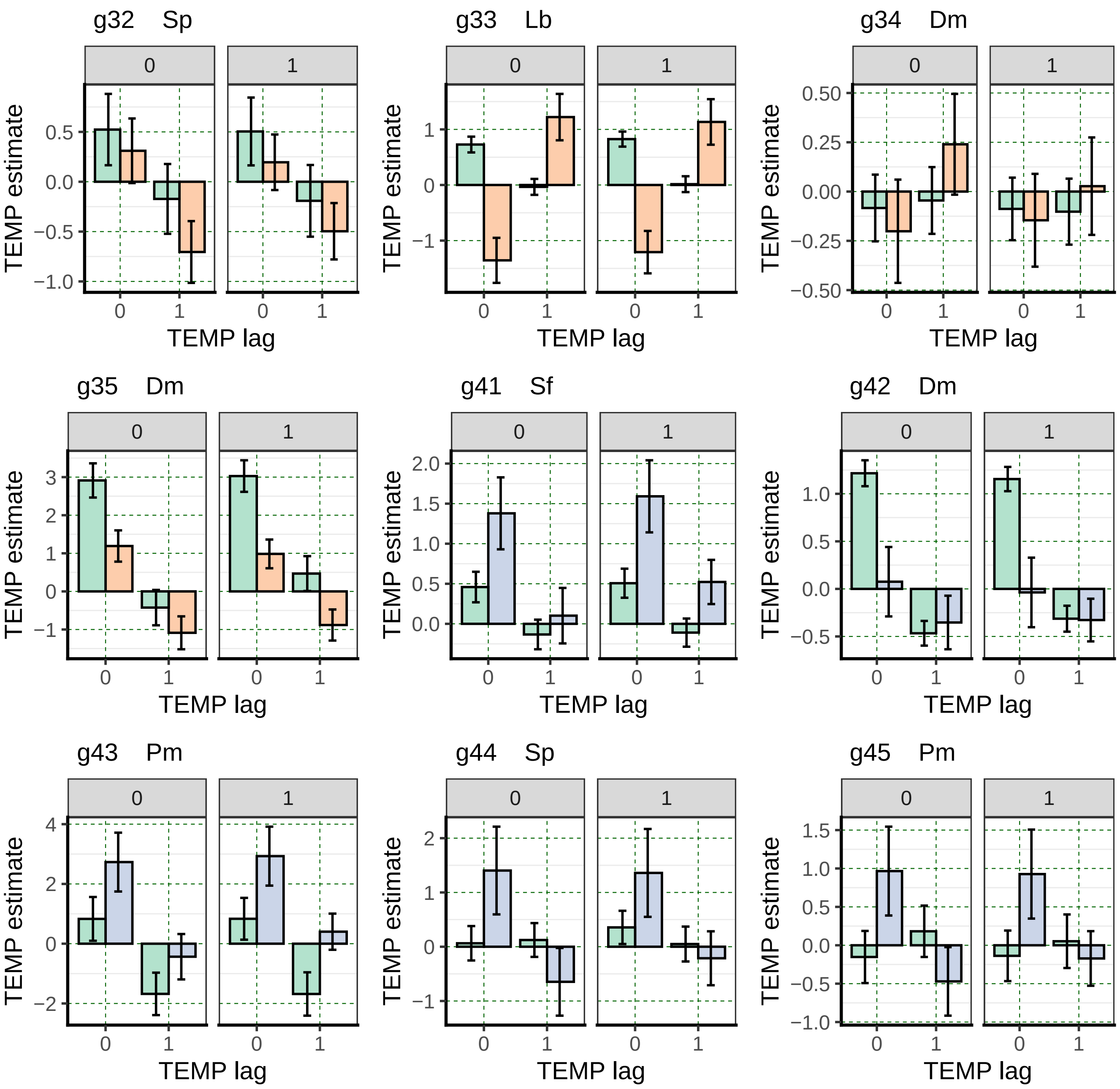

Supplement: S6 Appendix — (PDF) [file pone.0270140.s006.pdf]
